# Inhibition of RACGAP1 sensitizes triple-negative breast cancer cells to ferroptosis by regulating CPT1A-dependent fatty acid metabolism

**DOI:** 10.1186/s13046-025-03568-4

**Published:** 2025-12-24

**Authors:** Zhike Zhou, Ye Hua, Jun Ma, Wenqiang Cong, Rui Zhan, Kexin Kang, Lu Wang, Hongyi Wei

**Affiliations:** 1 Department of Geriatrics, The First Hospital of China Medical University, Shenyang, liaoning 110001 PR China; 2 Department of Urology, Shengjing Hospital of China Medical University, No. 36, Sanhao Street, Heping District, Shenyang, Liaoning 110004 China; 3 Department of Neurobiology, School of Life Sciences, China Medical University, Shenyang, China; 4 Department of Oncology, Shengjing Hospital of China Medical University, No. 36, Sanhao Street, Heping District, Shenyang, Liaoning 110004 China

**Keywords:** Triple-negative breast cancer, Fatty acid oxidation, Ferroptosis, RACGAP1, MAZ, CPT1A

## Abstract

**Background:**

Triple-negative breast cancer (TNBC) is highly aggressive tumor with limited therapeutic options. Studying the molecular mechanisms underlying TNBC is necessary to address the unmet need in novel therapeutic targets. TNBC is demonstrated to have robust fatty acid (FA) metabolism activity, and recent studies proposed the linkage of FA metabolism with ferroptosis sensitivity. Hence, this study aimed to explore the targets that may regulate FA metabolism to sensitize TNBC cells to ferroptosis.

**Methods:**

RNA-sequencing data in The Cancer Genome Atlas (TCGA) and four microarray datasets in Gene Expression Omnibus (GEO) database were analyzed to identify key target *RACGAP1*, followed by a series of functional experiments to explore the exact role of *RACGAP1* in two TNBC cell lines (human MDA-MB-231 and mouse 4T1) and Xenograft tumor model. Dual-luciferase and chromatin immunoprecipitation (ChIP) assay was utilized to verify the binding of *RACGAP1* and *MAZ*. RNA sequencing on 4T1 cells transfecting with sh-NC and sh-*RACGAP1* was performed to validate the actions of *RACGAP1*.

**Results:**

*RACGAP1* was highly expressed in breast cancer, and associated with poor prognosis and ferroptosis activity. *RACGAP1* silencing could inhibit tumor cells survival and promote ferroptosis, and such anti-tumor activity could be blocked by ferroptosis inhibitors. RNA-sequencing analysis suggested that *RACGAP1* silencing could inhibit FA metabolism activity, which was further confirmed by metabolic analysis and the reduced level of ATP, triglyceride and FA oxidation. *CPT1A* overexpression reversed such changes, indicating that the regulation of *RACGAP1* on FA metabolism was *CPT1A*-dependent. Activation of FA metabolism activity or *CPT1A* overexpression blocked the ferroptosis sensitivity induced by *RACGAP1* silencing. Transcription factor *MAZ* was identified to directly up-regulate the expression of *RACGAP1*.

**Conclusion:**

Inhibition of *RACGAP1* sensitized TNBC cells to ferroptosis by inhibiting *CPT1A*-mediated FA metabolism. Targeting *RACGAP1* might be feasible strategy for TNBC management.

**Graphical Abstract:**

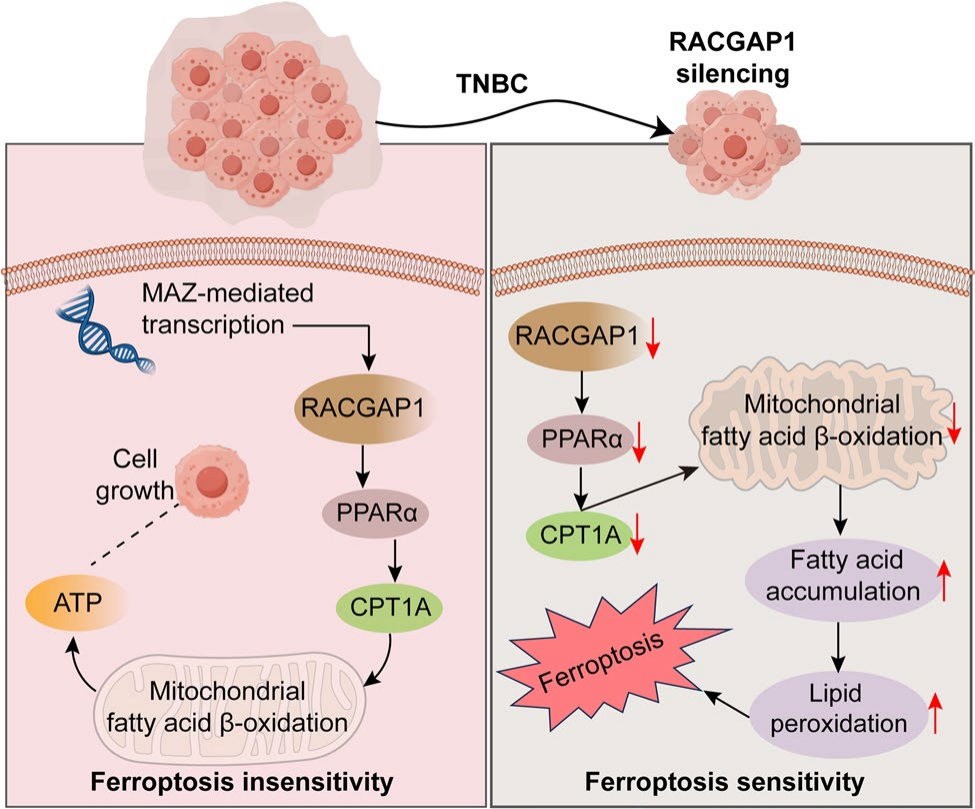

**Supplementary Information:**

The online version contains supplementary material available at 10.1186/s13046-025-03568-4.

## Highlights

1. *RACGAP1* is identified as an oncogenic gene in breast cancer.

2. *RACGAP1* silencing sensitizes TNBC cells to ferroptosis and inhibits fatty acid metabolism activity.

3. Transcription factor *MAZ* regulates the expression of *RACGAP1* in TNBC.

4. Regulation of *RACGAP1* on fatty acid metabolism depends on *CPT1A*.

5. *RACGAP1* regulated *CTP1A* expression by activating the PPARα.

**Supplementary Information:**

The online version contains supplementary material available at 10.1186/s13046-025-03568-4.

## Introduction

 Breast cancer is the most common tumor among women, with 2,308,897 newly diagnosed cases in 2022 worldwide, which is also responsible for the main cause of cancer-related deaths in women, causing 665,684 deaths in 2022 [1]. Although the complicated pathogenesis and various clinical manifestations of breast neoplasms pose remarkable difficulties to its prevention and therapy, the establishment of diverse delicate molecular subtypes improves such situations [2]. Triple-negative breast cancer (TNBC) represents the most malignant subtype of breast cancer (accounts for approximately 15–18%), with the highest rates of recurrence and mortality [3–5]. Approved targeted therapy is lacked for patients with TNBC, and cytotoxic chemotherapy remains the primary treatment regime for those despite of the resistance [6], and recurrence occurs in 30% of patients who have good initial response to chemotherapy, within 5 years after treatment [7]. Such situation highlights the requirements for developing novel therapeutic strategies in the context of TNBC.

Lipid metabolism reprogramming is a core factor in tumorigenesis and development, and targeting lipid metabolism has emerged as an available strategy in cancer therapy [8]. Tumor cells frequently facilitate de novo synthesis of fatty acids (FA) to support their biosynthetic and energy needs [9], and evade therapeutic attacks by taking advantage of the functions of lipids in various important cellular life activities [10]. Maladjusted lipid metabolism is linked to tumor progression and resistance to treatments [11–13]. Fatty acid synthase (FASN), a crucial enzyme needed for FA biosynthesis, is frequently highly expressed in tumors [14]. Repression of FASN can efficiently repress cell cycle progression and growth of tumor cells [15, 16]. Several FASN inhibitors have gained continuous attentions as potential anti-cancer drugs [17, 18]. In TNBC, expression of *FASN* is commonly observed, and FASN inhibitors display therapeutic benefit in both chemoresistant and sensitive TNBC preclinical models [7]. TNBC tumor is demonstrated to exhibit robust lipid metabolism activity, and targeting FA binding protein 4-mediated lipid metabolism can inhibit the progression of TNBC [19]. Up-regulation of fatty acid oxidation (FAO) intermediates is observed in MYC overexpressed TNBC, and inhibition of FAO can reduce energy metabolism and repress tumor growth of this TNBC tumor subtype [20]. RGCC-driven oxidative phosphorylation and FAO are demonstrated as key targets for the therapy of TNBC lung metastasis [21]. These findings emphasize the crucial roles of FA metabolism in TNBC.

Ferroptosis is an iron-dependent cell death process driven by lipid peroxidation that is facilitated by the accumulation of polyunsaturated FA (PUFA) [22, 23]. Under normal circumstances, PUFAs are oxidized by lipoxygenases and then rapidly reduced by glutathione peroxidase 4 (*GPX4*) and its cofactor glutathione (GSH) [24, 25]. Nevertheless, the inhibition of *GPX4* or the depletion of GSH may cause the accumulation of lipid peroxides in cells, thus resulting in the lipid peroxidation-induced cell death, that is ferroptosis [25, 26]. Considering this, increasing attentions have focused the relationships across lipid metabolism and ferroptosis sensitivity [23, 27, 28]. Various compounds or genes such as Fatty-Acid-Coenzyme A Ligase, Long-Chain 4 (*ACSL4*) associated with lipid metabolism can modulate ferroptosis in tumor cells [29], highlighting the associations of lipid metabolism and ferroptosis sensitivity.

In this study, based on several transcriptome datasets, we identified Rac GTPase Activating Protein 1 (*RACGAP1*) as an oncogenic gene in breast cancer. Highly expression of *RACGAP1* has been observed in several human tumors, which is frequently associated with worse overall survival of patients [30, 31]. In breast cancer, overexpression of *RACGAP1* was frequently linked to a high tendency of nipple invasion, lymph node/distant metastasis, and an advanced TNM stage and worse survival outcomes [32, 33]. However, the exact roles of *RACGAP1* in breast cancer, particularly in TNBC is largely unclear. In this study, we found that expression of *RACGAP1* was associated with both ferroptosis activity and FA metabolism. Therefore, we proposed that *RACGAP1* might be a novel target that may regulate FA metabolism to sensitize TNBC cells to ferroptosis. Results of our study demonstrated the oncogenic role of *RACGAP1* and its regulation on ferroptosis sensitivity, which was mediated by upstream transcription factor MYC associated zinc finger protein (*MAZ*). Further functional experiments suggested that Inhibition of *RACGAP1* sensitizes triple-negative breast cancer cells to ferroptosis by regulating carnitine palmitoyltransferase 1 A (*CPT1A*)-dependent FA metabolism. Finding of this study will contribute us to understand the dysregulation and the prognosis associations of *RACGAP1* in breast cancer in clinic.

## Methods

### Public datasets acquisition and bioinformatics analysis

Gene expression data for breast cancer in The Cancer Genome Atlas (TCGA-BRCA) was downloaded from the UCSC Xena web portal (https://xenabrowser.net/datapages/). Besides, four microarray datasets for breast cancer, including GSE10810, GSE21422, GSE54002 and GSE134359, were also downloaded from the Gene Expression Omnibus (GEO, https://www.ncbi.nlm.nih.gov/geo/) database. The detailed information of these public datasets such as platform and sample size was summarized in Table 1.

Differential expression analysis between tumor and normal control in TCGA-BRCA was conducted utilizing DESeq2 package (version 1.42.1), and in other four microarray datasets was conducted utilizing Limma package (version 3.58), followed by Benjamini-Hochberg multiple testing corrections. Genes with adjusted *P* < 0.05 and |log_2_FC|>1 were selected as differentially expressed genes (DEGs). Univariate Cox survival was conducted utilizing Survival package (version 3.5–8.5) for the overlapped DEGs to explore their prognostic value. Gene set for ferroptosis was obtained based on KEGG pathway (hsa04216). With this gene set as enrichment reference, ferroptosis score was quantized by gene set variation analysis (GSVA) based on the TCGA-BRCA dataset. Pearson correlation analysis was employed to investigate the associations of key genes with ferroptosis. Transcription factors were predicted utilizing R package TF-Target Finder (version 0.1.0), and the binding sites of transcription factors with target genes were analyzed utilizing JASPAR website (https://jaspar.elixir.no/analysis).

**Table 1:** The detailed information of the used datasets

Data Set	Platforms	Type	Sample size	Usage
TCGA-BRCA	Illumina	mRNA	1097 BRCA 113 Normal	Differential analysis
GSE10810	GPL570	mRNA	31 BRCA 27 Normal	Differential analysis
GSE21422	GPL570	mRNA	5 BRCA 5 Normal	Differential analysis
GSE54002	GPL570	mRNA	417 BRCA 16 Normal	Differential analysis
GSE134359	GPL17586	mRNA	74 BRCA 12 Normal	Differential analysis

### Cell culture and treatments

Two TNBC cell lines, including human MDA-MB-231 and mouse 4T1, as well as human normal mammary epithelial cells MCF-10 A were obtained from the Guangdong Bohui Biotechnology Co., LTD (China). These cells were cultured in dulbecco’s modified eagle medium (DMEM) medium by an additional adding of 10% fetal bovine serum (FBS, #16140071, Gibco) and 1% penicillin-streptomycin (#SV30010, Hyclone) under the condition of 37 ℃ and 5% CO_2_.

For cells treatment, ferroptosis activators: erastin (#S80804), iFSP1 (#S89092) and SAS (#B82669); ferroptosis inhibitors: ferrostatin-1 (#S81461) and alpha-tocopherol (#Y28485); CPT1A activator C75 (#S84163) and Baicalin (#A10010); 5-LOX inhibitor zileuton (#T27633) were purchased from Shanghai Yuanye Biotechnology Co., LTD (China). Ferroptosis activators, erastin (1, 2, 5, 10, 20, 50 and 100 µM), iFSP1 (0.01, 0.1, 1, 10 and 100 µM) or SAS (0, 200, 400, 600, 800 and 1000 µM) were added to cells for 24 h of treatment [34]. CPT1A activator C75 (80 µM) [35], Baicalin (100 µM) [35], or 5-LOX inhibitor zileuton (20µM) [36] were added to cells for 3 h of treatment. Ferroptosis inhibitors: ferrostatin-1 (0.5µM) and alpha-tocopherol (5µM) were added to cells for 24 h of treatment. ROS scavengers N-acetyl-L-cysteine (NAC, #HY-B0215) and PPARα activator GW7647 (#HY13861) were purchased from MedChemExpress (MCE, USA). NAC (1.5 mM) [37] was added to cells for 48 h of treatment, and GW7647 (600 nM) [38] was added to cells for 24 h of treatment.

### Cell transfection

Lentiviral vector system was employed to inhibit *RACGAP1*, CCCTC-Binding Factor (*CTCF*), *MAZ* and Sp1 Transcription Factor (*SP1*) or overexpress *RACGAP1*, *CPT1A* and *MAZ* in TNBC cells. Sequences of shRNAs are listed in Table S1, while coding sequences of *RACGAP1*, *CPT1A* and *MAZ* was obtained from NCBI. Specifically, pLVX-shRNA2-Puro vectors carrying shRNAs (sh-*RACGAP1*, sh-*CTCF*, sh-*MAZ* and sh-*SP1*) and corresponding NC sequences and/or pCDH-CMV-MCS-EF1-T2A-Neo vectors carrying mature overexpression sequences (oe-*RACGAP1*, oe-*CPT1A* and oe-*MAZ*) and corresponding empty vectors (Vector) were transfected into 293 T cells together with lentiviral packaging plasmids (pMDLg/pRRE: pVSV-G: pRSV-Rev = 5:3:2) with the aids of HighGene reagent, followed by collecting and concentrating lentivirus particles 48 h of transfection. Afterwards, TNBC cells that seeded into 6-well plate (2 × 10^5^/mL) were infected with lentivirus (1 × 10^8^ TU/mL) when the cells at 70–90% fusion degree. After 72 h for infections, stable transfected cells were selected with the aids of 2.5 g/mL puromycin and/or neomycin.

### RNA extraction and qRT-PCR

Total RNAs were isolated from tissue samples and cells by Trizol reagent, and were reversely transcribed into complementary DNA (cDNA). PCR amplification was then carried out with the aids of SYBR Green PCR Master Mix under reaction conditions: 95 °C for 10 min and 40 cycles of 95 °C for 12 s and 60 °C for 40 s. The used primers are listed in Table S2.

### Western blot

Total proteins were isolated following cells lysis, and were quantified with the aids of bicinchoninic acid (BCA) assay (#PC0020, Solarbio). Proteins were electrophoretically separated, transferred onto polyvinylidene fluoride (PVDF, # FFP24, Beyotime) membranes and then were blocked. Afterwards, primary antibodies: RACGAP1 (#ab97315, Abcam), CPT1A (#ab220789, Abcam), MAZ (#ab85725, Abcam), caspase3 (#ab32351, Abcam), Poly(ADP-Ribose) Polymerase 1 (PAPR, #ab191217, Abcam), Mixed lineage kinase domain like pseudokinase (MLKL, #DF7412, Affinity), phosphorylated MLKL (p-MLKL, #AF7420, Affinity), peroxisome proliferator activated receptor alpha (PPARα, #ab233078, Abcam) and glyceraldehyde-3-phosphate dehydrogenase(GAPDH, #ab181602, Abcam), co-incubation with the membranes were carried out overnight at 4 °C, followed by incubation with secondary antibodies for 60 min at dark. Subsequently, the blotting bands were visualized with the aid of enhanced chemiluminescence (ECL) reagent (#34579, Pierce) followed by film exposure.

### CCK-8 assay

Cell viability was measured by a cell counting kit-8 (CCK-8) kit (#C0037, Beyotime). TNBC cell lines were seeded into 96-well plates (2000 cells/well) for 24 h of incubation. Then, 10 µL CCK-8 solution was added to each well and incubated with the cells for 2 h. Cell viability was determined by measuring the absorbance at 450 nm utilizing a microplate reader.

### Flow cytometry (FCM)

Cell samples were digested with 0.25% ethylene diamine tetraacetic acid (EDTA)-free trypsin. For apoptosis detection, cells were resuspended in binding buffer, and were double-stained using Annexin V- fluorescein isothiocyanate (FITC) and propidium iodide in accordance with the handbook of Annexin V-FITC apoptosis kit (#C1062M, Beyotime). Subsequently, cells were analyzed utilizing a flow cytometer and CELL Quest software. Besides, utilizing BODIPY-C11 (#HY-D1301, MCE) as the probe, lipid peroxidation was measured by FCM. Briefly, 100 µL 5 µM Bodipy-C11 working solution was added to the culture medium for 30 min incubation at 37 °C. After three times of washing, green fluorescence was detected and quantified by FCM and FlowJo_V10. Besides, cells were incubated with 100 µL work solution of the BODIPY 558/568 C12 dye (#HY-138226, MCE) for 30 min, and incubated with work solution of Mito-Tracker Green (Mitogreen, #C1048, Beyotime), followed by FCM analysis to evaluate lipid droplets deposition and mitochondria number in cells, respectively. BODIPY, a lipophilic dye, was used to stain lipid droplets, providing morphological information of neutral lipids in the cell. Mito-Tracker Green is a mitochondria-specific green fluorescent probe, can be used for specific fluorescence staining of mitochondria in living cells.

### Commercial kit assays

Intracellular malondialdehyde (MDA), GSH, tissue iron and adenosine triphosphate (ATP) level was determined utilizing malondialdehyde assay kit (#BC0025, Solarbio), the reduced glutathione assay kit (#BC1175, Solarbio), the Iron Assay Kit (#BC4355, Solarbio), and the ATP assay kit (#BC0300, Solarbio), respectively, in accordance with the manufacturer’s handbook. Lactate dehydrogenase (LDH) release is regarded as an important indicator of cell membrane integrity and is widely used in cytotoxicity detection. In this study, LDH release was determined with the aids of LDH Cytotoxicity Assay Kit (#C0016, Beyotime), which quantified the LDH activity using colorimetry by measuring the absorbance at 490 nm. To measure the triglyceride level, an Amplex Red Triglyceride Assay Kit (#S0219S, Beyotime) was employed, which determined the triglyceride content utilizing absorbance detection based on the probe Amplex Red. Level of FA oxidase was determined using enzyme-linked immunosorbent assay (ELISA) kit (#XEY-36286) obtained from the Shanghai Xinweiyu Biotechnology Co., Ltd.

### Transmission electron microscopy (TEM)

TNBC cells were fixed by 2.5% glutaraldehyde, followed by dehydration by a series of concentrations alcohol and acetone. Following penetration, cells were embedded to prepare sections, and were stained by 2% sodium acetate and lead citrate. The cellular morphological changes were examined under TEM (#HT7800, HITACHI) in accordance with the manufacturer’s handbook.

### Detection of labile iron pool (LIP)

Intracellular LIP was measured by means of Calcein-AM (calcein-acetoxymethyl ester) method. Cells were washed by PBS after digesting with trypsin, and then incubated with Calcein-AM (0.05 µM) for 15 min at 37 °C. Following washing by PBS, cells were incubated with deferiprone (DFO, 100 µM) for 1 h at 37 °C or without treatment, followed by fluorescence analysis utilizing FCM. Calcein was excited at 488 nm, and fluorescence was measured at 525 nm. The LIP level was determined by the difference of mean cellular fluorescence with and without DFO incubation.

### Live/dead assay

Calcein-AM/PI kit (#B-CHK103, Biogradetech) was employed for visualizing the alive or dead state of cells. Specifically, after digesting with trypsin, cells were collected and resuspended (1 × 105 cells/mL) in Calcein AM/PI staining solution. Following 15 min of incubation in the dark, survival status of cells was detected with the aids of a fluorescence microscope (EX/EM = 494/517 nm for alive cells in green; and EX/EM = 535/617 nm for dead cells in red).

### Reactive oxygen species (ROS) detection

ROS Assay Kit with CM-H2DCFDA (#S0035M, Beyotime), a kit using CM-H2DCFDA as fluorescence probe for detecting ROS, was utilized to monitor the generation of ROS. Briefly, cells were collected and resuspended in serum-free medium, and then CM-H2DCFDA working solution (10 µM) was added to cells for 30 min of incubation in the dark. After twice of washing, cells were detected using a fluorescence microplate reader.

### Evaluation of OCR and SRC

Oxygen consumption rate (OCR) was measured utilizing OCR Fluorometric Assay Kit (#E-BC-F068, Elabscience) as per the manufacturer’s manual. TNBC cells were seeded in 96-well plates (4 × 10^4^ cells/well) for 3–4 hrs to allow adherence to the plate for 24 h. Afterwords, extracellular O_2_ consumption reagent and fluorescent dye were added to each well, and the high sensitivity oil was added to limit diffusion of oxygen into the assay medium. Next, OCR was measured utilizing on a fluorescence plate reader at 1.5 min interval for 90 min (Ex/Em = 380/650 nm). Spare Respiratory Capacity (SRC) was calculated by the difference between maximal and basal OCR.

### Dual-luciferase assay

Promoter of human *RACGAP1* was amplified utilizing PCR, which was then cloned into the pGL3 basic vector (Promega). The used primers for amplification of *RACGAP1* were listed in Table S3. After that, we utilized the Lipofectamin™ 3000 (Invitrogen) to co-transfect the pcDNA3.1/PCDNA3.1-MAZ plasmid with sequences of wild-type (WT) or mutant-type (MUT) binding sites (BS1-BS5) of *RACGAP1* into 293 T cells. Following 48 h of incubation, luciferase activity was detected at a dark environment utilizing the Pierce™ Cypridina-Firefly Luciferase Dual Assay Kit (#16184, Thermo). The site-directed mutagenesis for binding sites was generated using the QuickMutation™ site-directed mutagenesis kit (#D0206M, Beyotime). Briefly, the paired primers for five binding sites (BS1-BS5) were designed, and the sequences were listed in Table S3. The site-directed mutagenesis reaction system was prepared according to the manufacturer’s manual (including 36 µL Nuclease-Free Water, 5 µL10X BeyoFusion™ Buffer (with Mg), 2 µL primers, 5 µL dNTP mix, 1 µL plasmid and 1 µL BeyoFusion™ DNA Polymerase), and then PCR amplification was performed. Afterwards, DpnI digestion was conducted by adding 1 µL of DpnI to the PCR reaction system for 5 min of incubation at 37 °C, followed by transformation and cloning identification.

### Chromatin Immunoprecipitation (ChIP)

CHIP-PCR was conducted to detect the binding of MAZ to the RACGAP1 promoter. Briefly, after fixing in 1% formaldehyde, cells were lysed, and the cell lysates were prepared into chromatin fragments of 500 bp by ultrasound. Immunoprecipitation was carried out by incubating with MAZ and IgG antibodies at 4 °C on a constant temperature shaker for 2 h. The bound DNA was eluted by adding Proteinase K, and purified utilizing phenol-chloroform-isoamyl alcohol, followed by PCR. The used primer sequences were listed in Table S4. Electrophoresis on 2% agarose gel was conducted to detect the products of PCR.

### Xenograft tumor model

Female BALB/c nude mice (4 weeks, *n* = 24) were adaptively fed for one week in a controlled environment with a temperature of 22–25℃ and a humidity of 55%±5% as well as a 12-hour light/dark cycle. After this, MDA-MB-231 cells, and MDA-MB-231 cells transfected with NC, sh-*RACGAP1* and sh-*RACGAP1* + oe-*CPT1A*, respectively, were suspended in 200 µL of Matrigel (5 mg/mL) PBS, and then were injected subcutaneously into one flank. Tumor size was recorded every four days when it reached 50 mm^3^. At day 20, mice were sacrificed to collect tumor tissues for subsequent experiments after anesthesia by intraperitoneally injection of 150 mg/kg phenobarbital. All animal experiments were approved by Shengjing Hospital of China Medical University (Ethics No. 2025PS1274K), which were performed in accordance with ARRIVE guidelines, and approved by the laboratory animal ethics committee.

### Histological staining

The collected tumor tissues were fixed by 4% paraformaldehyde, embedded in paraffin, and were prepared into sections for histological staining. Paraffin sections were dewaxed and hydrated routinely. To observe tissue pathological feature and fat, the sections were stained by hematoxylin and Eosin (H&E) and Oil red O staining in accordance with the handbook of the Hematoxylin and Eosin Staining Kit (#C0105S, Beyotime) and Oil Red O Staining Kit (#C0157S, Beyotime). For immunohistochemistry, after antigen retrieval and blocking endogenous peroxidase activity, sections were incubated overnight at 4 °C with antibodies against Ki67 (1:200; #AF0198, Affinity) and GPX4 (1:200; #ab219592, Abcam) followed by incubation with secondary antibodies (1:2000). Next, sections were stained by 3–3′-diaminobenzidine and re-stained by hematoxylin, and were observed under an optical microscope following dehydration and permeation treatments. For immunofluorescence, after drying, fixing and permeating, the sections were blocked by 5% BSA and were incubated overnight at 4 °C with 4-hydroxynonenal (4-HNE) antibody (1: 200; #7631, CST), followed by incubation with secondary antibodies and DAPI staining. After sealing, the sections were examined under laser confocal microscopy.

### RNA sequencing (RNA-seq)

For RNA-seq, 4T1 cells transfecting with sh-NC and sh-RACGAP1 were utilized (*n* = 3 in each group). Briefly, following total RNAs extraction, the RNA was inversely transcribed to generate the cDNA, which was then utilized for library construction. Following purification, the library was sequenced on NovaSeq 6000 (Illumina, Inc., San Diego, CA, USA) platform.

### Statistical analysis

All experimental data was presented as mean ± standard deviation and was statistically analyzed and graphed utilizing GraphPad 7.0 software. Differences within two groups were detected utilizing unpaired t-test (two tailed), and among three groups were detected utilizing analysis of variance (ANOVA) coupled with Tukey’s multiple comparisons. Statistical significance existed when *P* < 0.05.

## Results

### *RACGAP1* is up-regulated in breast cancer and associated with poor prognosis

Based on RNA-seq data of TCGA-BRCA, we screened 5048 DEGs in breast cancer compared to normal controls, including 3028 up-regulated and 2020 down-regulated genes. Besides, there were 1406 DEGs (593 up- and 813 down-) in GSE10810 dataset, 1910 DEGs (926 up- and 884 down-) in GSE21422 dataset, 1460 DEGs (617 up- and 843 down-) in GSE54002 dataset and 3610 DEGs (1827 up- and 1783 down-) in GSE134359 dataset, respectively. The up- and down-regulated genes among five datasets were merged, and 190 overlapped genes (95 up- and 95 down-) were screened (Fig. S1A, Table S5). The prognostic value of these 190 genes was investigated based on TCGA cohort, and the detailed information for this cohort was summarized in Table S6. Among these 190 genes, univariate Cox survival based on TCGA-BRCA cohort revealed 15 genes that significantly associated with prognosis of patients, and further multivariate Cox identified five prognostic genes, including *RAD54B*, *RACGAP1*, *SLC7A5*, *EMP1* and *IGFBP6* (Fig. S1B). *RACGAP1* was up-regulated in breast cancer, and its high expression was associated with worse survival outcome of patients, identified as risk factor in breast cancer. Both mRNA and protein expression of *RACGAP1* was also confirmed to be up-regulated in two cell lines, MDA-MB-231 and 4T1, compared with those in normal mammary epithelial cell MCF-10 A (Fig. S1C). We quantified the activity score of ferroptosis for each sample in TCGA cohort (*n* = 1210), and further correlations analysis between ferroptosis activity and genes expression (Fig. S1D) suggested that *RACGAP1* expression was negatively correlated with the activity of ferroptosis (*r*=–0.11). The linkages of *RACGAP1* with ferroptosis had not been reported yet, and therefore, *RACGAP1* was selected as a target gene to investigate its roles in ferroptosis in breast cancer.

### *RACGAP1* Silencing caused breast cancer cells death can be blocked by ferroptosis inhibitors

**Fig. 1:**
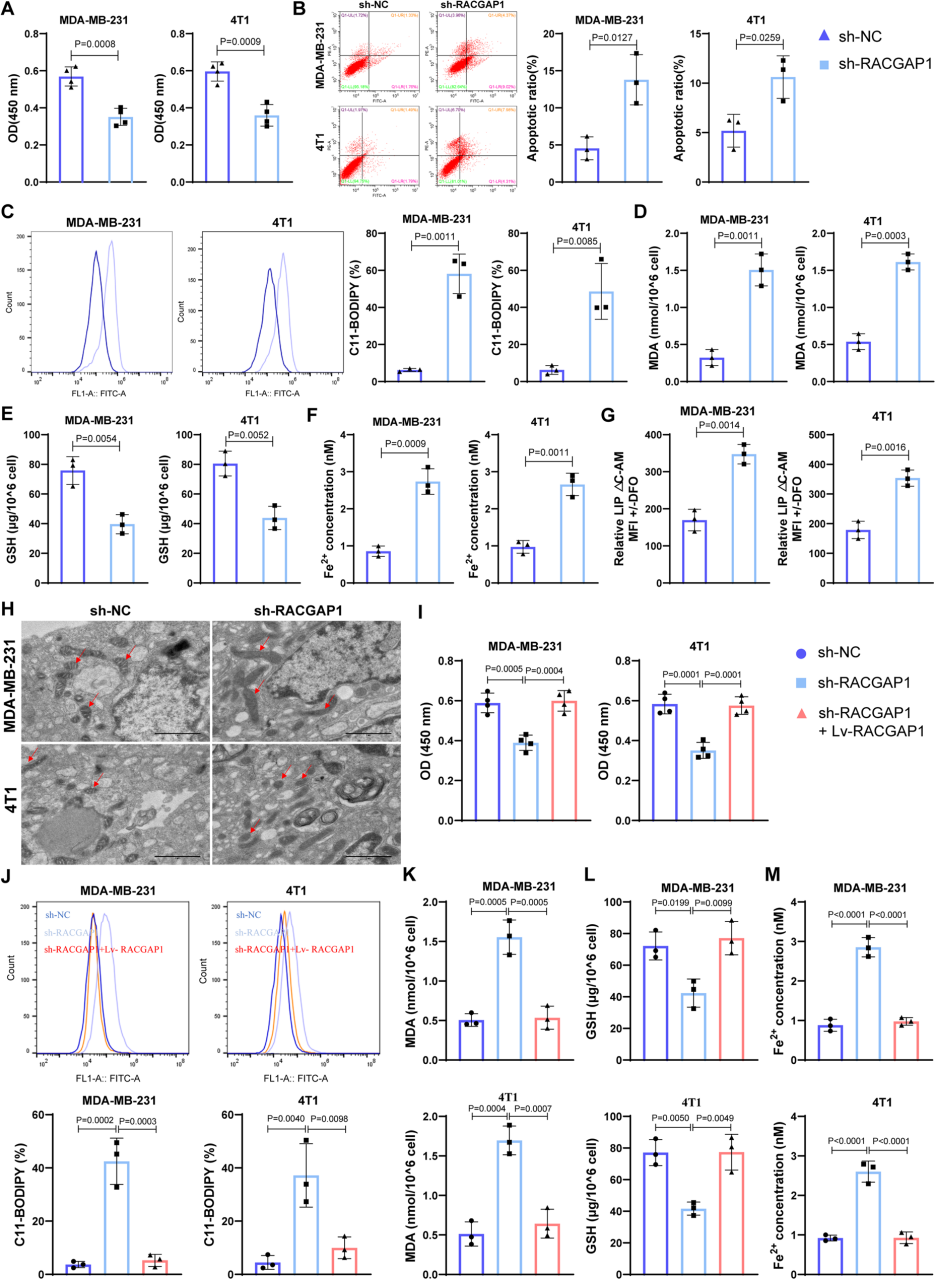
*RACGAP1* silencing caused breast cancer cells ferroptosis. **A**, CCK-8 assay (*n* = 4) showing cell viability after *RACGAP1* silencing; **B**, FCM analysis (*n* = 3) showing cell apoptosis after *RACGAP1* silencing; **C**, FCM analysis (*n* = 3) showing the lipid peroxidation level (oxidized C11-BODIPY) after *RACGAP1* silencing; **D**-**G**, level of intracellular MDA (**D**), GSH (E), Fe^2+^ (**F**) and labile iron pool (LIP, G) after *RACGAP1* silencing (*n* = 3); H, ferroptosis ultrastructural changes after *RACGAP1* silencing observed under transmission electron microscope (*n* = 3); I, CCK-8 assay (*n* = 4) showing cell viability after *RACGAP1* silencing and overexpression; **J**, FCM analysis (*n* = 3) showing the lipid peroxidation level (oxidized C11-BODIPY) after *RACGAP1* silencing and overexpression; **K**-**M**, level of intracellular MDA (**K**), GSH (**L**) and Fe^2+^ (**M**) after *RACGAP1* silencing and overexpression (*n* = 3)

To explore the roles of *RACGAP1* in breast cancer, we successfully silenced or overexpressed its expression in both MDA-MB-231 and 4T1 cells (Fig. S1E). *RACGAP1* silencing markedly inhibited the cell viability (Fig. 1A), whereas induced cell apoptosis (Fig. 1B) of these two cell lines. Meanwhile, *RACGAP1* silencing seemed to enhance ferroptosis of TNBC cells. Ferroptosis is an iron-dependent cell death process driven by lipid peroxidation [22]. FCM analysis for oxidized C11-BODIPY, a well-recognized ferroptosis marker, suggested that *RACGAP1* silencing markedly elevated the lipid peroxidation level than NC group (Fig. 1C). Also, the amount of MDA (lipid peroxidation marker) was increased while the amount of GSH (primary antioxidant in cells) was reduced after *RACGAP1* silencing (Fig. 1D-E), indicating that TNBC cells with *RACGAP1* silencing exhibited observably higher oxidative damage. Meanwhile, *RACGAP1* silencing cells exhibited a significantly increase on the amount of Fe^2+^ (Fig. 1F), and cellular LIP (free iron contents in cells, Fig. 1G). TEM analysis showed mitochondrial atrophy and reduction of mitochondrial cristae (Fig. 1H), which provided typical ferroptosis ultrastructural changes. Further overexpression of *RACGAP1* in the *RACGAP1*-silenced cells significantly counteracted the effects of *RACGAP1* silencing on cell viability (Fig. 1I), lipid peroxidation level of oxidized C11-BODIPY (Fig. 1J) and MDA (Fig. 1K), antioxidant GSH (Fig. 1L) and the amount of Fe^2+^ (Fig. 1M). These results confirmed that *RACGAP1* was a driver in the malignant progression of TNBC.

**Fig. 2:**
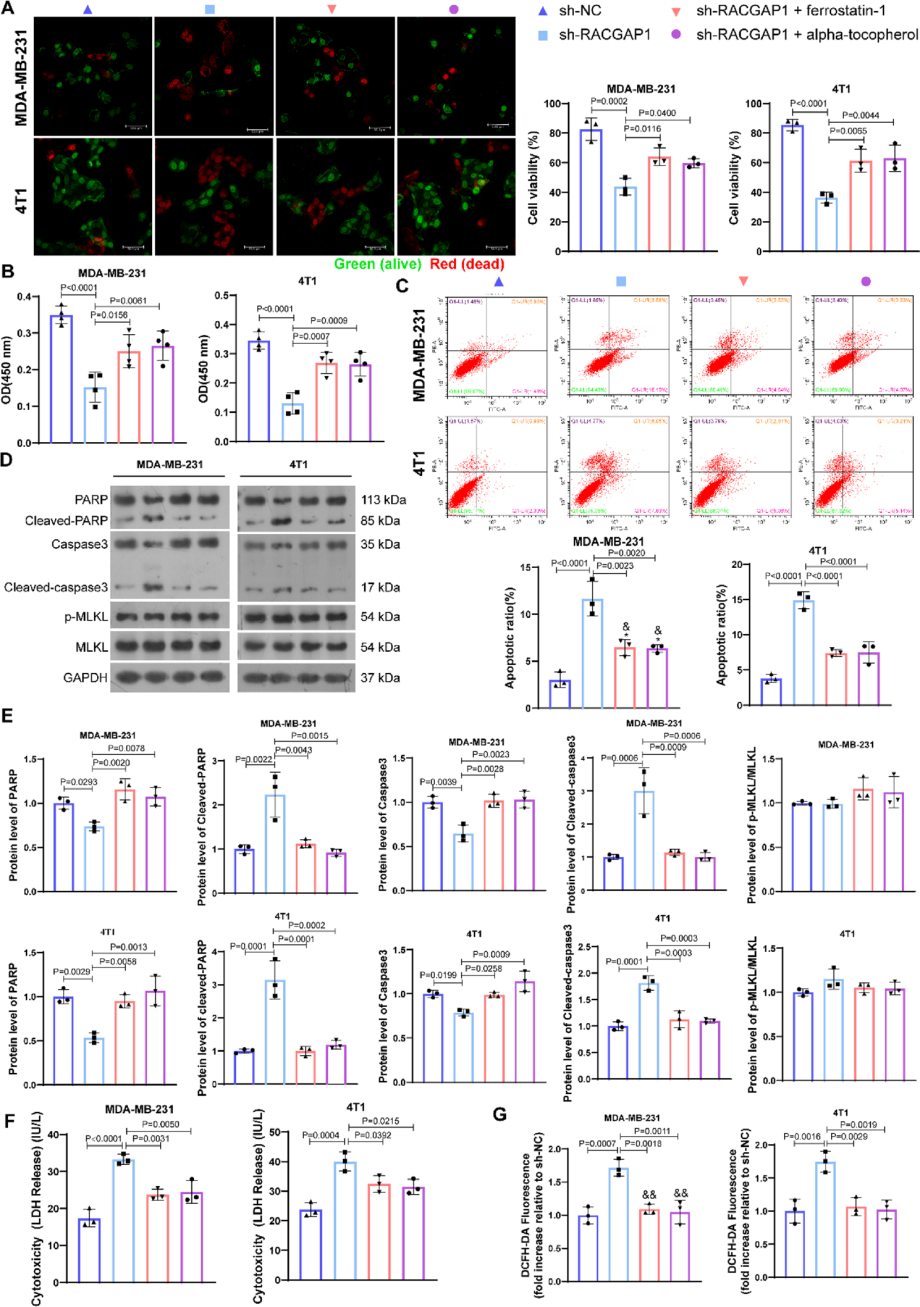
*RACGAP1* silencing caused breast cancer cells death could be blocked by ferroptosis inhibitors. **A**, representative image (left) and quantification (right) of live/dead assay showing the alive (green) and dead (red) cells after RACGAP1 silencing and/or ferroptosis inhibition (*n*=3); **B**, CCK-8 assay (*n*=4) showing the cell viability after RACGAP1 silencing and/or ferroptosis inhibitors treatments; **C**, representative image (upper) and quantification (below) of FCM showing the cell apoptosis after RACGAP1 silencing and/or ferroptosis inhibitors treatments (n=3); **D**-**E**, protein bands (D) and quantification (E) of western blot showing the expression of RARP, cleaved-RARP, caspase3, cleaved-caspase3 and p-MLKL/MLKL (n=3); **F**, LDH release after RACGAP1 silencing (*n*=3); **G**, ROS generation detected by DCFH-DA fluorescence after RACGAP1 silencing and/or ferroptosis inhibitors treatments (*n*=3)

**Fig. 3:**
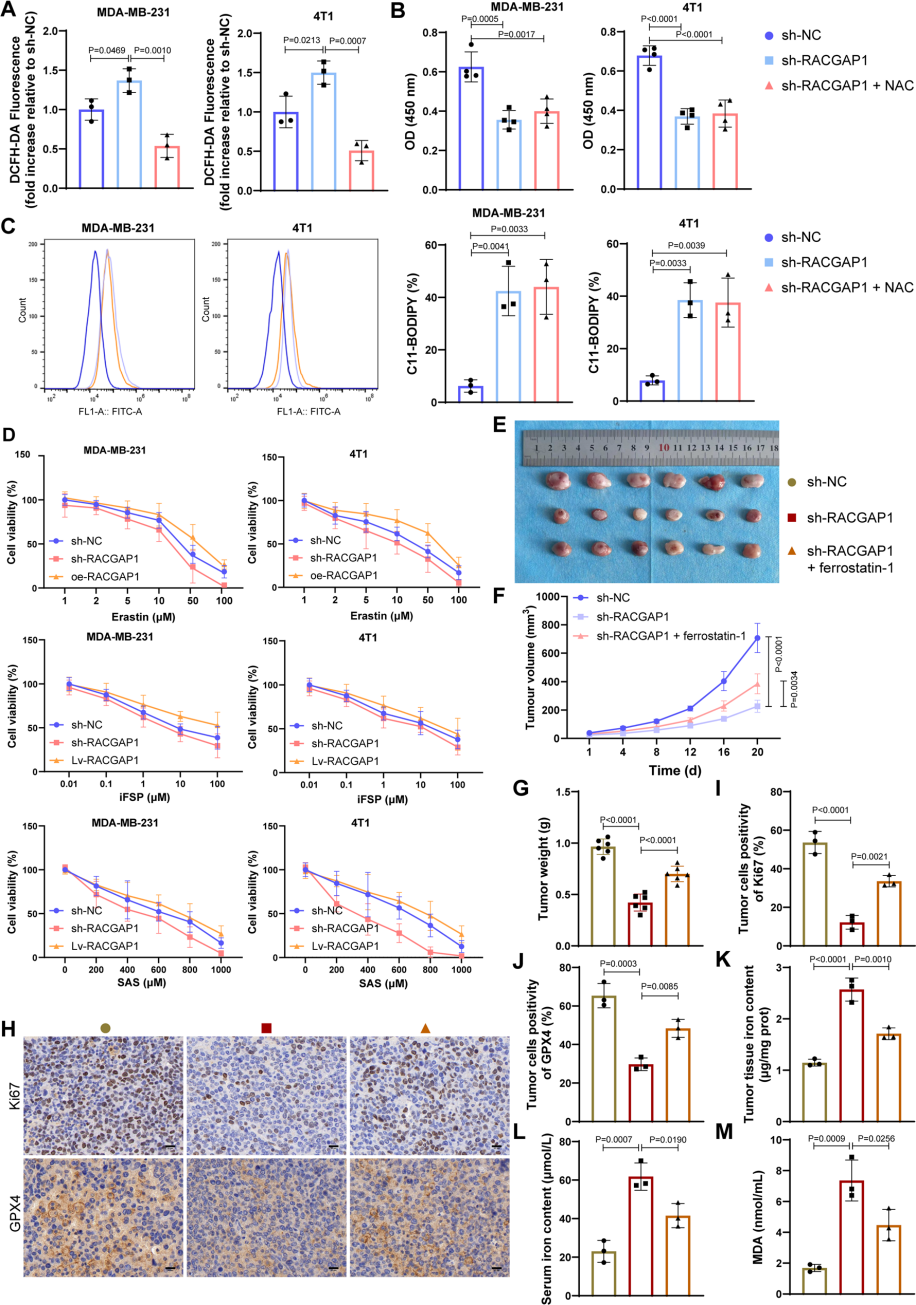
*RACGAP1* silencing could induce ferroptosis both in vitro and in vivo. **A**, ROS generation detected by DCFH-DA fluorescence after *RACGAP1* silencing and/or NAC treatment (*n* = 3); **B**, CCK-8 assay (*n* = 4) showing the cell viability after *RACGAP1* silencing and/or NAC treatment; **C**, representative image (left) and quantification (right) of FCM analysis showing the lipid peroxidation level (oxidized C11-BODIPY) after *RACGAP1* silencing and/or NAC treatment (*n* = 3); **D**, CCK-8 assay (*n* = 4) showing the dose-dependent cytotoxicity of MDA-MB-231 and 4T1 cells when grown in different concentrations of SAS, erastin and iFSP for 24 h; E, images of the dissected tumor tissue from tumor-bearing mice in each group (*n* = 6); F-G, tumor volume (**F**) and tumor weight (**G**) of mice in each group (*n* = 6); **H**-**J**, representative images (**H**) and quantification of Ki67 (**I**) and GPX4 (**J**)expression in tumor tissue determined by immumohistochemical staining (*n* = 3); **K**, iron content in tumor tissue of mice in each group (*n* = 3); **L**, serum iron content of mice in each group (*n* = 3); **M**, Serum MDA level of mice in each group (*n* = 3)

We speculated that *RACGAP1* silencing induced cell death was partly achieved by increasing ferroptosis, and to confirm this assumption, ferroptosis inhibitors, ferrostatin-1 and alpha-tocopherol, were utilized. Both ferrostatin-1 and alpha-tocopherol significantly reversed the reduced cell viability (Fig. 2A-B, Fig. S2A) and enhanced cell apoptosis (Fig. 2C) caused by *RACGAP1* silencing. Level of cleaved-caspase 3 and cleaved-PARP was increased after *RACGAP1* silencing, such increases could be counteracted after additional treatments by ferroptosis inhibitors, ferrostatin-1 and alpha-tocopherol (Fig. 2D-E). Level of MLKL phosphorylation showed no significant changes among groups (Fig. 2D-E). Besides, LDH release assay indicated that there was more LDH release in sh-RACGAP1 group, and such increase could be partly blocked by ferrostatin-1 or alpha-tocopherol treatment (Fig. 2F). Both ferrostatin-1 and alpha-tocopherol also markedly reduced the generation of ROS caused by *RACGAP1* silencing (Fig. 2G). To test whether lipid peroxidation caused by *RACGAP1* silencing was ROS-dependent, we treated the cells using ROS scavengers NAC (1.5 mM) for 48 h [37]. NAC treatment significantly reduced the ROS generation caused by *RACGAP1* silencing (Fig. 3A), but showed no significant influences on cell viability (Fig. 3B) and the lipid peroxidation level (Fig. 3C). This suggested that *RACGAP1* silencing caused lipid peroxidation-dependent cell death. Cells were further treated by different concentrations of ferroptosis activators, erastin, iFSP1 or SAS, and dose-dependent cytotoxicity was determined by CCK-8 assay (Fig. 3D). As a results, MDA-MB-231 and 4T1 cells in sh-*RACGAP1* group showed lowest cells viability than those in NC group and oe-*RACGAP1* group, while the cell viability of oe-*RACGAP1* group was similarly to the NC group. This suggested that *RACGAP1* silencing enabled TNBC cells more sensitive to ferroptosis. In vivo experiments confirmed this conclusion. Specifically, *RACGAP1* silencing markedly reduced the tumor weight and volume (Fig. 3E-G) as well as the expression of proliferation marker Ki67 (Fig. 3H-I) of the tumor-bearing mice, while such anti-tumor effects of *RACGAP1* silencing were partly counteracted by ferroptosis inhibitor ferrostatin-1 treatment. As for ferroptosis markers, *RACGAP1* silencing markedly reduced the expression of *GPX4* (Fig. 3H and J), while increased the tumor tissue iron content (Fig. 3K), serum iron content (Fig. 3L) and serum peroxidation MDA level (Fig. 3M). Such changes could be partly counteracted by ferroptosis inhibitor ferrostatin-1 treatment.

### *RACGAP1* Silencing regulates the activity of FA metabolism

**Fig. 4:**
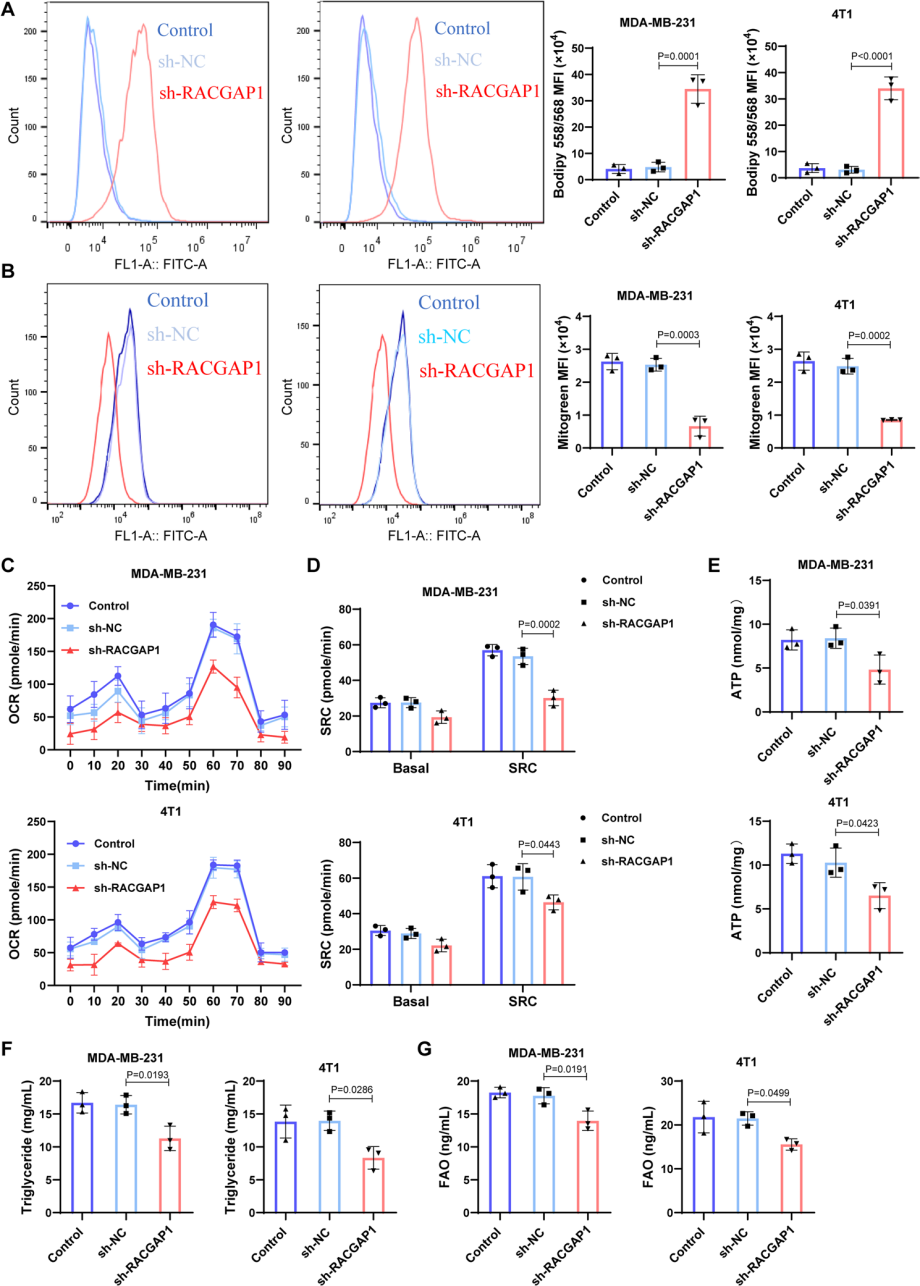
*RACGAP1* silencing reduced the activity of fatty acid metabolism. **A**, Bodipy 558/568 staining showing the changes of lipid droplets deposition in MDA-MB-231 and 4T1 breast cells after *RACGAP1* silencing (*n* = 3); **B**, FCM analysis showing the changes of Mitogreen-labeled mitochondria in MDA-MB-231 and 4T1 breast cells after *RACGAP1* silencing (*n* = 3); **C**-**D**, changes of oxygen consumption rates (OCR, **C**) and spare respiratory capacity (SRC, **D**) in MDA-MB-231 and 4T1 breast cells after *RACGAP1* silencing (*n* = 3); **E**, ATP production in MDA-MB-231 and 4T1 breast cells after *RACGAP1* silencing (*n* = 3); **F**, level of triglyceride in MDA-MB-231 and 4T1 breast cells after *RACGAP1* silencing (*n* = 3); **G**, level of fatty acid oxidase (FAO) in MDA-MB-231 and 4T1 breast cells after *RACGAP1* silencing (*n* = 3)

Reprogramming of FA metabolic is proposed as a considerable driver of cancer progression, particularly in breast cancer [39, 40]. To explore whether there was association between *RACGAP1* and FA metabolism, we performed RNA-seq on 4T1 cells transfecting with sh-NC and sh-*RACGAP1* (Fig. S2A). Enrichment analysis revealed that the dysregulated genes between groups were mainly associated with synthesis and metabolism-related pathway, as well as cell death related pathways, such as ferroptosis (Fig. S2B-C). Gene set enrichment analysis indicated that activity of FA metabolism pathway was down-regulated in *RACGAP1* silencing group (Fig. S2D). Meanwhile, expression of FA metabolism-related genes was also reduced along with the reduced expression of *RACGAP1* in sh-*RACGAP1* group, and there were positive correlations between these genes (Fig. S2E-F). These results suggested that *RACGAP1* also regulated FA metabolism in breast cancer. Therefore, we investigated the actions of *RACGAP1* silencing on FA metabolism activity. FAs metabolism converts nutrients into metabolic intermediates for synthesis of membranes and signaling molecules and energy production via its β-oxidation [18]. Compared to control groups, there was more lipid droplets deposition in sh-*RACGAP1* group than corresponding controls (Fig. 4A). Besides, cells transfecting with sh-*RACGAP1* presented less mitochondria than controls (Fig. 4B). Metabolic analysis indicated that cells in *RACGAP1* silencing group possessed reduced mitochondrial OCR and observably decreased SRC (Fig. 4C-D), accompanied by a reduced ATP level (Fig. 4E). These results implied a low mitochondrial oxidative phosphorylation after *RACGAP1* silencing. Meanwhile, level of triglyceride and FA oxidase was also down-regulated after *RACGAP1* silencing (Fig. 4F-G). Besides, we found that *RACGAP1* silencing markedly down-regulated the expression of genes related to FAO (*CPT1A*, *CPT1B*, *ACADV1* and *HADH*), FA synthesis (*FASN*, *ACSl1*, *ACACA*, *SCD1* and *DGAT1*) and lipolysis (*LP1*, *LIPE* and *ABHD5*) (Fig. S3). These results suggested that *RACGAP1* silencing reduced the activity of FA metabolism.

### Regulation of *RACGAP1* on FA metabolism depends on *CPT1A*

**Fig. 5:**
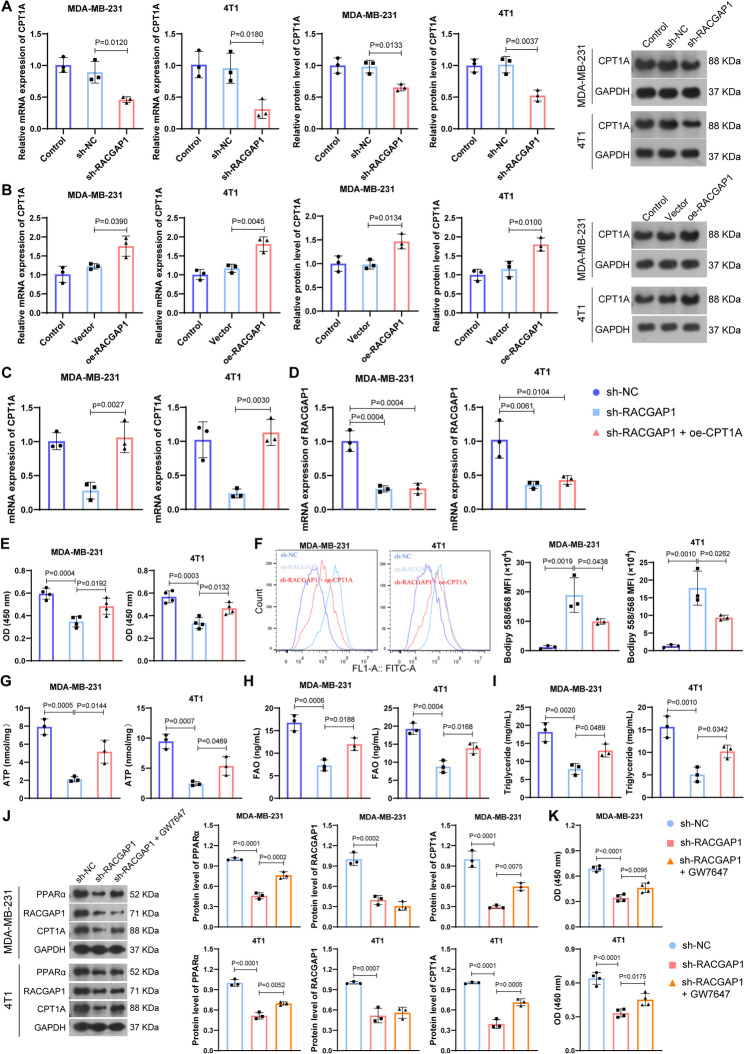
Regulation of *RACGAP1* on fatty acid metabolism depends on *CPT1A*. **A-B**, the mRNA and protein expression of *CPT1A* in MDA-MB-231 and 4T1 breast cells after *RACGAP1* silencing (**A**) or *RACGAP1* overexpression (**B**) (*n* = 3); **C**, the mRNA expression of *CPT1A* in MDA-MB-231 and 4T1 breast cells after *RACGAP1* silencing and/or *CPT1A* overexpression (*n* = 3); **D**, the mRNA expression of *RACGAP1* in MDA-MB-231 and 4T1 breast cells after *RACGAP1* silencing and/or *CPT1A* overexpression (*n* = 3); **E**, CCK-8 assay (*n* = 4) showing the cell viability of cells after *RACGAP1* silencing and/or *CPT1A* overexpression; **F**, Bodipy 558/568 staining showing the changes of lipid droplets deposition in MDA-MB-231 and 4T1 breast cells after *RACGAP1* silencing and/or *CPT1A* overexpression (*n* = 3); **G**-**I**, the ATP production (**G**), level of fatty acid oxidase (FAO, **H**), and level of triglyceride (**I**) in MDA-MB-231 and 4T1 breast cells after *RACGAP1* silencing and/or *CPT1A* overexpression (*n* = 3); **J**, protein bands (left) and quantification (right) of PPARα, RACGAP1 and CPT1A in MDA-MB-231 and 4T1 breast cells after *RACGAP1* silencing and/or GW7647 (PPARα activator) treatment (*n* = 3); **K**, CCK-8 assay (*n* = 4) showing the cell viability of cells after *RACGAP1* silencing and/or GW7647 (PPARα activator) treatment

CPT1A is a key enzyme involved in FA metabolism, which is well-recognized as rate-limiting enzyme in FAO, and its deficiency leads to reduced rate of FAO [41]. We found that *RACGAP1* silencing markedly reduced the expression of *CPT1A* in breast cancer cell lines (Fig. 5A), while *RACGAP1* overexpression enhanced the *CPT1A* expression (Fig. 5B). *RACGAP1* and *CPT1A* seemed to be co-expressed, similarly to the positive correlation between *RACGAP1* and FA metabolism activity. Positive correlations between *CPT1A* and *RACGAP1* expression confirmed the co-expression of these two (Fig. S1A). To validate whether *RACGAP1* regulated FA metabolism by targeting *CPT1A*, we successfully overexpressed *CPT1A* in breast cancer cells transfecting with sh-*RACGAP1* (Fig. 5C), and its overexpression exhibited no influences on the expression of *RACGAP1* (Fig. 5D). *CPT1A* overexpression partly blocked the decrease of sh-*RACGAP1* on cell viability (Fig. 5E). Besides, as expected, the higher level of lipid droplets deposition after *RACGAP1* silencing was reversed after *CPT1A* overexpression, manifested as less lipid droplets deposition (Fig. 5F). Meanwhile, the reduced level of ATP (Fig. 5G), FA oxidase (Fig. 5H) and triglyceride (Fig. 5I) after *RACGAP1* silencing was also markedly reversed after *CPT1A* overexpression. These findings suggested that *CPT1A* overexpression could offset the decrease of FA metabolism activity caused by *RACGAP1* silencing.

We further explored the possible mechanistic link between *RACGAP1* and *CPT1A*. Peroxisome proliferator-activated receptor alpha (*PPAR*α) is a nuclear receptor that serves as a metabolic sensor to regulate lipid homeostasis. CPT1A is a downstream target of *PPAR*α, and can be transcriptionally regulated by *PPAR*α [42, 43]. Transcriptome data suggested that expression of *PPAR*α was positively correlated with *RACGAP1* expression (Fig. S4B). Therefore, we speculated that *RACGAP1* regulated the expression of *CTP1A* by activating the *PPAR*α. As expected, expression of *PPAR*α was reduced after *RACGAP1* silencing, while its expression was increased after *RACGAP1* overexpression (Fig. S4C). PPARα activator GW7647 (600 nM) was used to activate *PPAR*α in *RACGAP1*-silenced cells, which showed no significant changes on *RACGAP1* expression, while significantly enhanced the expression of *CPT1A* (Fig. 5J). In addition, *PPAR*α activator GW7647 also partly blocked the decrease of sh-*RACGAP1* on cell viability (Fig. 5K). These results implied that *RACGAP1* regulated the expression of *CTP1A* by activating the *PPAR*α.

### Activation of FA metabolism activity blocks the ferroptosis sensitivity induced by *RACGAP1* Silencing

**Fig. 6:**
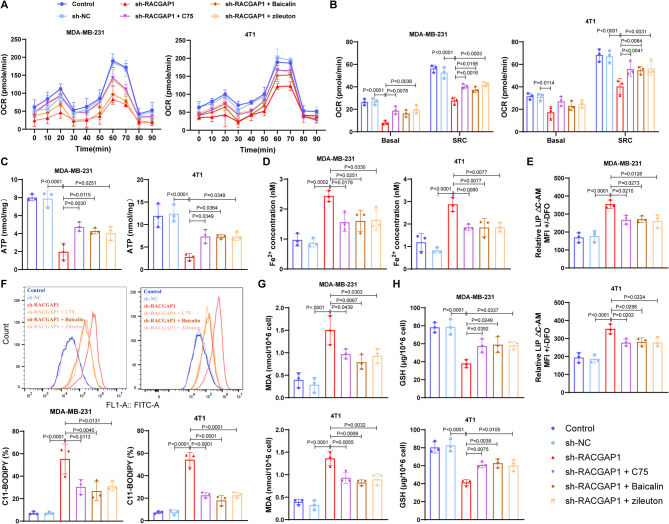
*RACGAP1* silencing induced breast cancer cells ferroptosis by inhibiting fatty acid metabolism. **A**-**B**, changes of oxygen consumption rates (OCR, **A**) and spare respiratory capacity (SRC, **B**) in MDA-MB-231 and 4T1 breast cells after treatments (*n* = 3); **C**, ATP production in MDA-MB-231 and 4T1 breast cells after treatments (*n* = 3); D-E, level of intracellular Fe^2+^ (**D**) and labile iron pool (LIP, **E**) in MDA-MB-231 and 4T1 breast cells after treatments (*n* = 3); **F**, FCM analysis showing the lipid peroxidation level (oxidized C11-BODIPY) after treatments (*n* = 3); **G**-**H**, level of intracellular MDA (**G**) and GSH (**H**) in MDA-MB-231 and 4T1 breast cells after treatments (*n* = 3)

Studies have demonstrated the close linkages between FA metabolism and the ferroptosis sensitivity [44–46]. Therefore, we assumed that *RACGAP1* deficiency–induced ferroptosis sensitivity was mediated by FA metabolism. To test this assumption, *CPT1A* activator (C75 and Baicalin) and 5-LOX inhibitor zileuton were utilized. Metabolic analysis indicated that the reduced mitochondrial OCR and SRC (Fig. 6A-B) as well ATP level (Fig. 6C) in *RACGAP1* silencing group were improved after treatment with C75, Baicalin and zileuton. This indicated that C75, Baicalin and zileuton treatment enhanced the activity of FA metabolism. Ferroptosis features were then observed, and the results indicated that the elevated Fe^2+^ content (Fig. 6D), intracellular LIP content (Fig. 6E), lipid peroxidation level (Fig. 6F) and the level of lipid peroxidative marker MDA (Fig. 6G) in *RACGAP1* silencing group were partly reversed after C75, Baicalin and zileuton treatment. Inversely, the reduced antioxidant GSH level (Fig. 6H) caused by *RACGAP1* silencing was markedly elevated after C75, Baicalin and zileuton treatment. These results implied that the enhanced ferroptosis sensitivity caused by *RACGAP1* silencing could be partly blocked by promoting the activity of FA metabolism.

### Transcription factor MAZ regulates the expression of *RACGAP1* in breast cancer

**Fig. 7:**
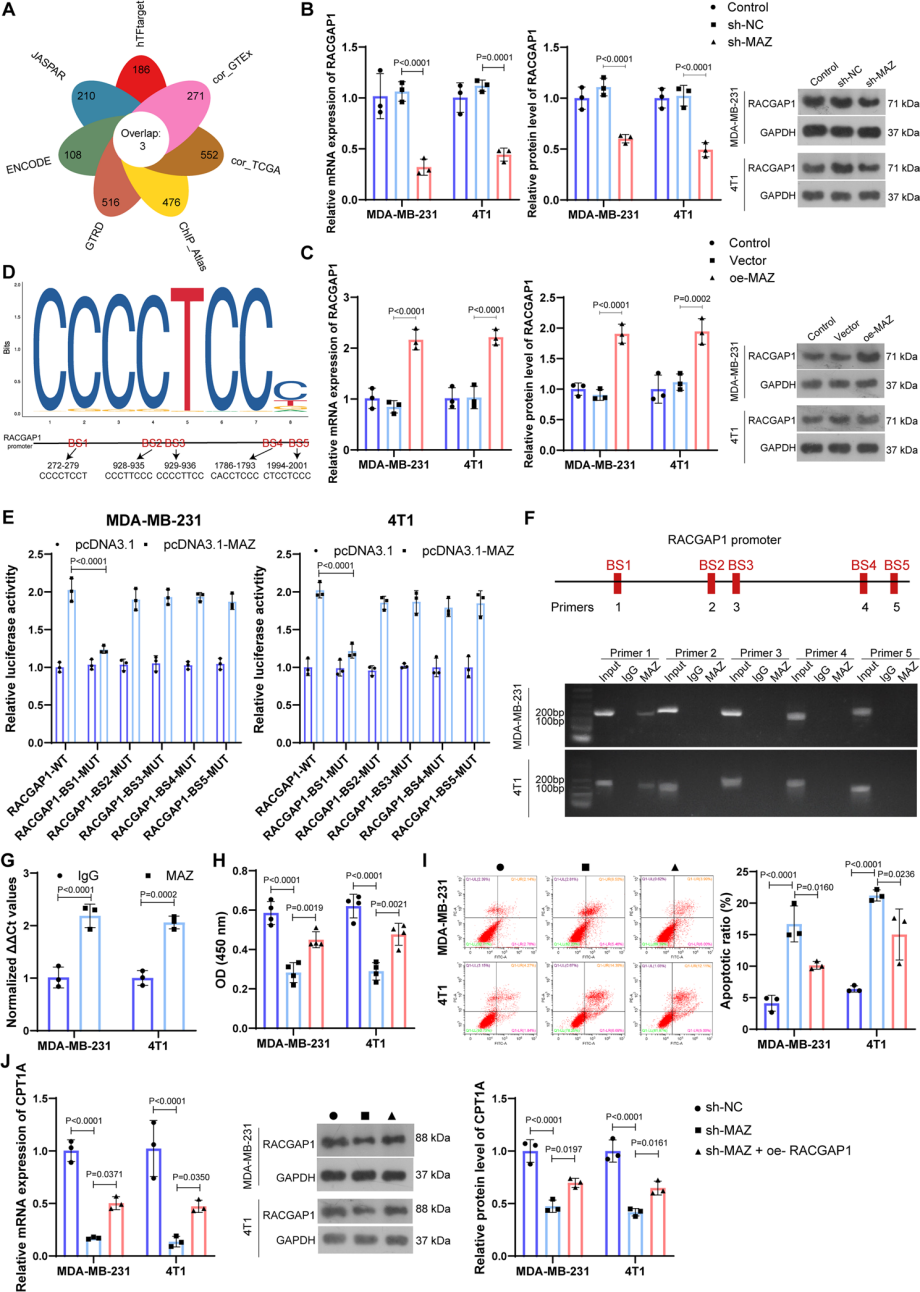
Transcription factor *MAZ* regulates the expression of *RACGAP1* in breast cancer. **A**, the predicted transcription factors for *RACGAP1* analyzed by TF-Target Finder; **B**-**C**, the mRNA and protein expression of *RACGAP* 1 in MDA-MB-231 and 4T1 breast cells after *MAZ* silencing (**B**) or *MAZ* overexpression (**C**) (*n* = 3); **D**, the binding motif and the five binding site sequences of *MAZ* binding to *RACGAP1* promoter analyzed by JASPAR database; **E**, dual-luciferase assay in MDA-MB-231 and 4T1 breast cells for validating the binding of *MAZ* to *RACGAP1* promoter by BS1 (*n* = 3); **F**, the agarose gel electrophoresis of CHIP-PCR products showing the binding of MAZ to *RACGAP1* promoter by BS1, IgG in this assay was utilized as negative control; **G**, ChIP assay was conducted utilizing IgG or MAZ antibodies for immunoprecipitation in MDA-MB-231 and 4T1 breast cells, followed by qPCR using primer corresponding to BS1 (*n* = 3); **H**, CCK-8 assay (*n* = 4) showing the cell viability of cells after *MAZ* silencing and/or *RACGAP1* overexpression; **I**, FCM analysis showing the apoptosis of cells after *MAZ* silencing and/or *RACGAP1* overexpression (*n* = 3); **J**, the mRNA and protein expression of *CPT1A* in MDA-MB-231 and 4T1 breast cells after *MAZ* silencing and/or *RACGAP1* overexpression (*n* = 3)

To interpret the dysregulation of *RACGAP1* in breast cancer, the upstream transcriptional regulation was investigated. TF-Target Finder is a web application that bridges multiple predictive tools (such as hTFtarget, FIMO_JASPAR and ENCODE) for decoding the interactions between transcription factor and target. Using TF-Target Finder, we predicted transcription factors targeting *RACGAP1* from seven related online tools, and three overlapped transcription factors were obtained, including CTCF, MAZ and SP1 (Fig. 7A). Subsequently, actions of these three transcription factors on the expression of *RACGAP1* were explored, and sh-*MAZ* exhibited prominent effects on the expression of *RACGAP1* (Fig. S5A-B). Specifically, at both mRNA and protein level, *MAZ* silencing and/or overexpression could effectively reduce and/or elevate the expression of RACGAP1 in MDA-MB-231 and 4T1 cell lines, respectively (Fig. S5C-D, Fig. 7B-C). Afterwards, we utilized the JASPAR database predict the sites of *MAZ* binding to *RACGAP1*, and five binding sites were obtained with relative profile score threshold > 0.85 (Fig. 7D), with BS1 showing a highest binding score of 0.973. Dual-luciferase assay was then conducted based on the co-transfection of pcDNA3.1-*MAZ* and constructs containing both the WT and MUT of binding sequence (BS1-BS5) of *RACGAP1*. As a result, luciferase activity was observably reduced when the BS1 binding sequence of *RACGAP1* was mutated, whereas the luciferase activity showed no significant changes in the MUT group of BS2-5 (Fig. 7E), indicating that MAZ could bind to *RACGAP1* promotor by the BS1 site. This was further confirmed by CHIP-PCR. In which, only the DNA fragments containing BS1 was markedly enriched in the chromatin that was precipitated with MAZ antibody in both MDA-MB-231 and 4T1 cells (Fig. 7F and G). Transcriptome data from clinical samples revealed that expression of both *MAZ* and *RACGAP1* was increased in BRCA tumor tissue than that in normal tissue, and there was significant positive correlation between *MAZ* and *RACGAP1* (Fig. S6). The biological function of *MAZ* was also explored in breast cancer cells (Fig. S5E). The results indicated that *MAZ* silencing markedly repressed the cell viability of MDA-MB-231 and 4T1 cells, while such repression was partly offset via *RACGAP1* overexpression (Fig. 7H). Similarly, *MAZ* silencing triggered more cell apoptosis, and such increase on cell apoptosis was partly blocked by *RACGAP1* overexpression (Fig. 7I). Besides, *MAZ* silencing could repress the expression of *CPT1A*, and such repression was partly counteracted when *RACGAP1* overexpressed (Fig. 7J). These results implied that MAZ might promote breast cancer progression by elevating the expression of *RACGAP1*.

### *RACGAP1* Silencing enhances breast cancer ferroptosis by targeting *CPT1A in vivo*

**Fig. 8:**
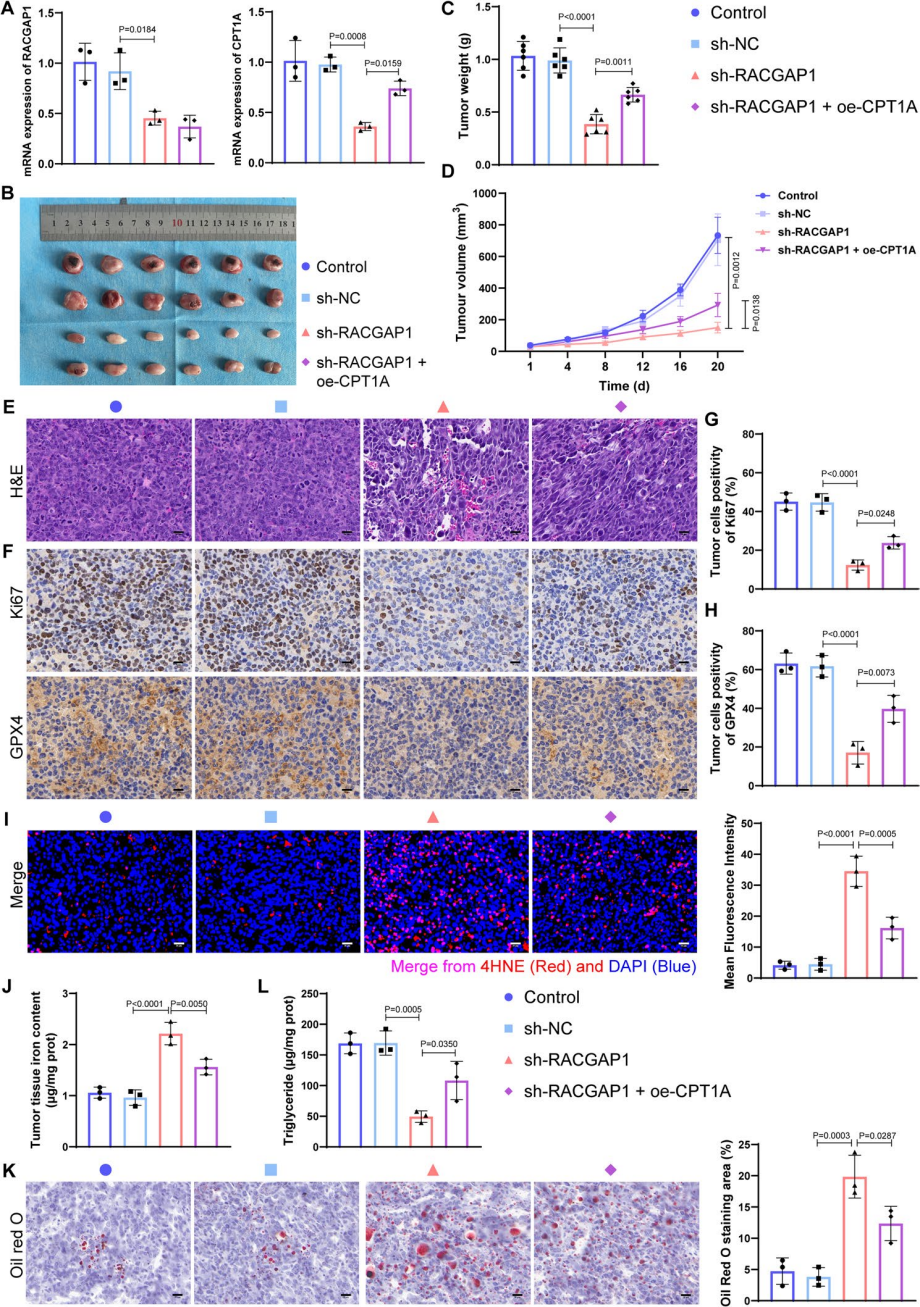
*RACGAP1 *silencing enhanced breast cancer ferroptosis by targeting *CPT1A in vivo*. **A**, the mRNA expression of *RACGAP1* (left) and *CPT1A* (right) in cells after *RACGAP1* silencing and/or *CPT1A* overexpression (*n* = 3); **B**, images of the dissected tumor tissue from tumor-bearing mice in each group (*n* = 6); **C**-**D**, tumor weight (**C**) and tumor volume (**D**) of mice in each group (*n* = 6); **E**, representative images of tumor tissue H&E staining (*n* = 3); **F**-**H**, representative images (**F**) and quantification of Ki67 (**G**) and GPX4 (**H**) expression in tumor tissue determined by immumohistochemical staining (*n* = 3); **I**, representative images (left) and quantification (right) of 4-HNE in tumor tissues determined by immunofluorescence (*n* = 3); **J**, iron content in tumor tissue of mice in each group (*n* = 3); **K**, representative images (left) and quantification (right) of lipid droplets in tumor tissues determined by oil red O staining (*n* = 3); **L**, level of triglyceride in tumor tissues of mice in each group (*n* = 3)

To verify the anti-tumor effect of sh-*RACGAP1* in vivo, we established xenograft tumor models by subcutaneously injecting MDA-MB-231 cells, and MDA-MB-231 cells transfected with NC, sh-*RACGAP1* and sh-*RACGAP1* + oe-*CPT1A*, respectively. Expression of *RACGAP1* and *CPT1A* was confirmed by qRT-PCR before injection (Fig. 8A). Mice in sh-*RACGAP1* possessed smallest tumor volume and tumor weight than control and sh-NC groups, indicating *RACGAP1* silencing could observably inhibit breast cancer growth in vivo. While such anti-tumor activity was partly offset by *CPT1A* overexpression (Fig. 8B-D). H&E staining revealed that there was more tumor cells necrosis in sh-*RACGAP1* group, and *CPT1A* overexpression weakened such necrosis caused by *RACGAP1* silencing (Fig. 8E). Consistently, there were less Ki67 (cell proliferation marker) positive staining in tumor tissue from mice in sh-*RACGAP1* group, which was enhanced after *CPT1A* overexpression (Fig. 8F-G). The sh-*RACGAP1* group showed lowest expression of *GPX4*, a glutathione peroxidase blocking ferroptosis by reducing the accumulation of lipid peroxidation, and *CPT1A* overexpression significantly enhanced the expression of *GPX4* (Fig. 8F and H). Besides, sh-*RACGAP1* group showed highest level of 4-HNE (Fig. 8I), a ferroptosis marker indicating cytotoxic lipid peroxidation, and highest iron content (Fig. 8J) in tumor tissue than control and sh-NC groups, while such changes were partly blocked by *CPT1A* overexpression. Oil red O staining indicated that there was a significant accumulation of lipid droplets in sh-*RACGAP1* group, and *CPT1A* overexpression partly offset such lipid droplets accumulation (Fig. 8K). Besides, *RACGAP1* silencing markedly inhibited the triglyceride level, while such inhibition was partly reversed after *CPT1A* overexpression (Fig. 8L). Altogether, these results suggested that *RACGAP1* silencing sensitizes breast cancer ferroptosis by regulating *CPT1A*-dependent FA metabolism.

## Discussion

In this study, we identified *RACGAP1* as an oncogene in TNBC, and its silencing could effectively inhibit the tumor growth and progression of TNBC by enhancing ferroptosis. Mechanically, based on RNA-seq analysis and functional experiments, we found that *RACGAP1* regulated the activity of FA metabolism in a *CPT1A*-dependent manner, thus modulating the sensitivity of TNBC cells to ferroptosis. Besides, MAZ was identified as an upstream transcription factor to regulate the expression of *RACGAP1*. Overall, the findings from our current study supported that targeting *RACGAP1* was an available strategy for TNBC management.

*RACGAP1* is a member of the Rho GTPase-activating proteins family that function as molecular switch in signal transduction by binding to the conformational changes caused by GTP [47]. The protein encoded by *RACGAP1* is a component of the centralspindlin complex, and is an essential protein for the formation of the myosin contraction ring during the cytokinesis [31]. *RACGAP1* negatively modulates the Rho-mediated signals by binding the active form of Rho GTPases and stimulating the hydrolysis of GTP, thus regulating various cellular processes [31]. Expression of *RACGAP1* has been reported to be highly elevated in multiple human tumors, and its expression is frequently linked to a poor prognosis [30, 31]. In breast cancer, overexpression of *RACGAP1* was frequently linked to a high tendency of nipple invasion, lymph node/distant metastasis, and an advanced TNM stage and worse survival outcomes [32, 33]. In terms of functions, overexpression of was found to facilitate tumor metastasis via modulating mitochondrial quality control [48]. However, the exact role of *RACGAP1* in TNBC is largely unclear. Consistently with these findings, our current study also identified *RACGAP1* as an oncogene in breast cancer, associated with poor survival in breast cancer. To illustrate the actions of *RACGAP1* in TNBC, we selected two TNBC cell lines, including human MDA-MB-231 and mouse 4T1. In both cell lines, *RACGAP1* was highly expressed, and its silencing could effectively repress TNBC cells survival, probably by enhancing ferroptosis.

Dysregulation of cells death contributes to the development of various human disorders, particularly cancers. Induction of cells death has proposed as feasible strategy for cancer management by considering that evading cell death is a hallmark of malignant tumor cells [49–51]. Ferroptosis is an iron-dependent cell death process involving oxidative damage of lipid molecules, which caused by the deregulation across antioxidant defenses (GSH) and the generation of peroxides such as ROS and MDA [52]. Inducing ferroptosis in tumor cells is a potential treatment strategy for TNBC [53, 54]. However, tumor cells may resist ferroptosis by regulating iron metabolism and the antioxidant system, thereby promoting tumor progression [55, 56]. In this study, *RACGAP1* expression was found to negatively correlated with ferroptosis activity based on RNA-seq analysis. *RACGAP1* silencing could enhance the sensitivity of TNBC cells to ferroptosis, evidenced by an elevated level of Fe^2+^, cellular LIP, oxidized C11-BODIPY (a well-recognized ferroptosis marker) and MDA (lipid peroxidation marker), while a reduced level of GSH (primary antioxidant in cells), as well as mitochondrial atrophy and reduction of mitochondrial cristae in TEM analysis. The anti-tumor activity of *RACGAP1* silencing was blocked by ferroptosis inhibitors, suggesting that *RACGAP1* could inhibit TNBC cells survival by sensitizing TNBC cells to ferroptosis.

Mitochondrial fatty acid β-oxidation (FAO) is the core process of FA metabolism. During this process, long-chain fatty acids can be transformed into fatty acids of appropriate length to meet the metabolic needs of the body [57, 58]. FAs can generate more energy via FAO when glucose supply is limiting [58]. Increasing studies have reported the significance of FAO in progression and chemoresistance of TNBC [4, 59, 60]. Our results indicated *RACGAP1* silencing could inhibit FA metabolism activity, which accompanied by a reduced level of FAO, and mitochondrial oxidative phosphorylation (mitochondrial OCR and ATP level). Such regulatory was achieved in a *CPT1A*-dependent manner, and *CPT1A* overexpression might partly block the effects of *RACGAP1* silencing on FA metabolism.

Carnitine palmitoyl transferase 1 (CPT1) is the rate-limiting enzyme for FAO in mitochondria [61]. There are three subtypes of CPT1, namely *CPT1A*, *CPT1B* and *CPT1C* [62]. Among them, *CPT1B* and *CPT1C* shows specific tissue-cell distributions, while *CPT1A* is widely present in various tissue and cells [62]. CPT1A is located on the outer mitochondrial membrane and catalyzes the synthesis of fatty acyl carnitine from long-chain fatty acyl CoA and carnitine. Under the action of CACT in the inner mitochondrial membrane, the latter passes through the inner mitochondrial membrane into the mitochondrial matrix, while transporting equal molecules of carnitine out of the mitochondria. The fatty acyl carnitine that enters the mitochondria is transformed into fatty acyl CoA under the action of CPT2 in the inner mitochondrial membrane. In the mitochondria, nicotinamide adenine dinucleotide and flavin adenine dinucleotide generated by FAO are oxidized by the respiratory chain to produce ATP [42, 62]. Expression of *CPT1A* was elevated in breast cancer, which was frequently linked to an advanced progression and a worse prognosis [63]. *CPT1A* deficiency could repress the aggressive growth and radioresistance of breast cancers by blocking FAO [64].

Myc-associated zinc finger protein (MAZ) is a transcription factor belonging to Myc family. Substantial evidences have demonstrated that abnormal expression levels of MAZ is involved in tumor growth and inflammatory progression processes [65, 66], which has been proposed as an important influencing factor in the occurrence and development of cancer. In TNBC, transcription factor MAZ was reported to directly regulate multiple assumed tumor-promoting genes, such as SIPL1 [67] and BCKDK [68] to accelerate progression of TNBC. For example, *MAZ* was reported to up-regulate the expression of BCKDK, a highly expressed gene in TNBC tumor tissues involving in promoting tumorigenesis [68]. Similarly to these studies, we found that highly expression of *MAZ* was associated with malignant progression in TNBC, which functioned as an upstream transcription factor to up-regulate the expression of *RACGAP1* in TNBC.

## Conclusion

In summary, the findings from this study highlighted the close linkage of FA metabolism with ferroptosis sensitivity, and the significance of *RACGAP1* in progression and treatment of TNBC. Both in vitro and in vivo experiments supported the oncogenic role of *RACGAP1* in TNBC, which regulated the ferroptosis sensitivity of TNBC through a *CPT1A*-mediated FA metabolism. MAZ was identified as upstream transcription factor to up-regulate the expression of *RACGAP1* in TNBC. Overall, this study suggested that *RACGAP1* might be an available therapeutic target for TNBC management, and provided promising candidates for further investigation.

## Supplementary Information

Supplementary Material 1

Supplementary Material 2

Supplementary Material 3

Supplementary Material 4

Supplementary Material 5

Supplementary Material 6

Supplementary Material 7

Supplementary Material 8

Supplementary Material 9

Supplementary Material 10

Supplementary Material 11

Supplementary Material 12

Supplementary Material 13

Supplementary Material 14

## Data Availability

The datasets used and/or analyzed during the current study are available from the corresponding author on reasonable request.

## Abbreviations

ACSL4: Fatty-Acid-Coenzyme A Ligase, Long-Chain 4
ChIP: Chromatin immunoprecipitation
CPT1: Carnitine palmitoyl transferase 1
DEGs: Differentially expressed genes
FA: Fatty acid
FAO: Fatty acid oxidation
FASN: Fatty acid synthase
FCM: Flow cytometry
GEO: Gene Expression Omnibus
GPX4: Glutathione peroxidase 4
GSH: Glutathione
H&E: Hematoxylin and Eosin
LIP: Labile iron pool
MAZ: Myc-associated zinc finger protein
MDA: Malondialdehyde
MUT: Mutant-type
OCR: Oxygen consumption rate
PPARα: Peroxisome proliferator activated receptor alpha
PUFA: Polyunsaturated FA
RACGAP1: Rac GTPase Activating Protein 1
RNA-seq: RNA sequencing
ROS: Reactive oxygen species
SRC: Spare Respiratory Capacity
TCGA-BRCA: The Cancer Genome Atlas-Breast Cancer
TEM: Transmission electron microscopy
TNBC: Triple-negative breast cancer
WT: Wild-type

## References

[1] Bray F, Laversanne M, Sung H, Ferlay J, Siegel RL, Soerjomataram I, et al. Global cancer statistics 2022: GLOBOCAN estimates of incidence and mortality worldwide for 36 cancers in 185 countries. CA Cancer J Clin. 2024;74(3):229–63. 38572751 10.3322/caac.21834

[2] Xiong X, Zheng LW, Ding Y, Chen YF, Cai YW, Wang LP, et al. Breast cancer: pathogenesis and treatments. Signal Transduct Target Ther. 2025;10(1):49. 39966355 10.1038/s41392-024-02108-4 PMC11836418

[3] Leon-Ferre RA, Goetz MP. Advances in systemic therapies for triple negative breast cancer. BMJ. 2023;381:e071674. 37253507 10.1136/bmj-2022-071674

[4] Lu R, Hong J, Fu T, Zhu Y, Tong R, Ai D, et al. Loss of OVOL2 in triple-negative breast cancer promotes fatty acid oxidation fueling stemness characteristics. Adv Sci. 2024;11(24):e2308945. 10.1002/advs.202308945 PMC11199980 38627980

[5] Asleh K, Riaz N, Nielsen TO. Heterogeneity of triple negative breast cancer: current advances in subtyping and treatment implications. J Exp Clin Cancer Res. 2022;41(1):265. 36050786 10.1186/s13046-022-02476-1 PMC9434975

[6] Yin L, Duan JJ, Bian XW, Yu SC. Triple-negative breast cancer molecular subtyping and treatment progress. Breast Cancer Res. 2020;22(1):61. 32517735 10.1186/s13058-020-01296-5 PMC7285581

[7] Giró-Perafita A, Palomeras S, Lum DH, Blancafort A, Viñas G, Oliveras G, et al. Preclinical evaluation of fatty acid synthase and EGFR inhibition in triple-negative breast cancer. Clin Cancer Res. 2016;22(18):4687–97. 27106068 10.1158/1078-0432.CCR-15-3133

[8] Terry AR, Hay N. Emerging targets in lipid metabolism for cancer therapy. Trends Pharmacol Sci. 2024;45(6):537–51. 38762377 10.1016/j.tips.2024.04.007 PMC11162322

[9] Currie E, Schulze A, Zechner R, Walther TC, Farese RV Jr. Cellular fatty acid metabolism and cancer. Cell Metab. 2013;18(2):153–61. 23791484 10.1016/j.cmet.2013.05.017 PMC3742569

[10] Xiao Q, Xia M, Tang W, Zhao H, Chen Y, Zhong J. The lipid metabolism remodeling: a hurdle in breast cancer therapy. Cancer Lett. 2024;582:216512. 38036043 10.1016/j.canlet.2023.216512

[11] Cao Y. Adipocyte and lipid metabolism in cancer drug resistance. J Clin Invest. 2019;129(8):3006–17. 31264969 10.1172/JCI127201 PMC6668696

[12] Singh MK, Han S, Kim S, Kang I. Targeting lipid metabolism in cancer stem cells for anticancer treatment. Int J Mol Sci. 2024;25(20):11185. 10.3390/ijms252011185. 10.3390/ijms252011185 PMC11508222 39456967

[13] Lin Y, Liang Z, Weng Z, Liu X, Zhang F, Chong Y. CRSP8-driven fatty acid metabolism reprogramming enhances hepatocellular carcinoma progression by inhibiting RAN-mediated PPARα nucleus-cytoplasm shuttling. J Exp Clin Cancer Res. 2025;44(1):93. 40069732 10.1186/s13046-025-03329-3 PMC11895297

[14] Zhang M, Yu L, Sun Y, Hao L, Bai J, Yuan X, et al. Comprehensive analysis of FASN in tumor immune infiltration and prognostic value for immunotherapy and promoter DNA methylation. Int J Mol Sci. 2022;23(24):15603. 10.3390/ijms232415603. 10.3390/ijms232415603 PMC9779179 36555243

[15] Fritz V, Fajas L. Metabolism and proliferation share common regulatory pathways in cancer cells. Oncogene. 2010;29(31):4369–77. 20514019 10.1038/onc.2010.182 PMC3004916

[16] Ni J, Shang Y, Wang WD, Wang C, Wang AM, Li GJ, et al. FASN inhibitors enhance Bestatin-Related tumor cell apoptosis through upregulating PEPT1. Curr Mol Pharmacol. 2023;16(7):771–86. 36411574 10.2174/1874467216666221121121549

[17] Wang H, Zhou Y, Xu H, Wang X, Zhang Y, Shang R, et al. Therapeutic efficacy of FASN inhibition in preclinical models of HCC. Hepatology. 2022;76(4):951–66. 35076948 10.1002/hep.32359 PMC9309180

[18] Röhrig F, Schulze A. The multifaceted roles of fatty acid synthesis in cancer. Nat Rev Cancer. 2016;16(11):732–49. 27658529 10.1038/nrc.2016.89

[19] Yu L, Wei W, Lv J, Lu Y, Wang Z, Cai C. FABP4-mediated lipid metabolism promotes TNBC progression and breast cancer stem cell activity. Cancer Lett. 2024;604:217271. 39306229 10.1016/j.canlet.2024.217271

[20] Camarda R, Zhou AY, Kohnz RA, Balakrishnan S, Mahieu C, Anderton B, et al. Inhibition of fatty acid oxidation as a therapy for MYC-overexpressing triple-negative breast cancer. Nat Med. 2016;22(4):427–32. 26950360 10.1038/nm.4055 PMC4892846

[21] Cheng S, Wan X, Yang L, Qin Y, Chen S, Liu Y, et al. RGCC-mediated PLK1 activity drives breast cancer lung metastasis by phosphorylating AMPKα2 to activate oxidative phosphorylation and fatty acid oxidation. J Exp Clin Cancer Res. 2023;42(1):342. 38102722 10.1186/s13046-023-02928-2 PMC10722681

[22] Zheng S, Guan XY. Ferroptosis. Promising approach for cancer and cancer immunotherapy. Cancer Lett. 2023;561:216152. 37023938 10.1016/j.canlet.2023.216152

[23] Lorito N, Subbiani A, Smiriglia A, Bacci M, Bonechi F, Tronci L, et al. *FADS1/2* control lipid metabolism and ferroptosis susceptibility in triple-negative breast cancer. EMBO Mol Med. 2024;16(7):1533–59. 38926633 10.1038/s44321-024-00090-6 PMC11251055

[24] Seiler A, Schneider M, Förster H, Roth S, Wirth EK, Culmsee C, et al. Glutathione peroxidase 4 senses and translates oxidative stress into 12/15-lipoxygenase dependent- and AIF-mediated cell death. Cell Metab. 2008;8(3):237–48. 18762024 10.1016/j.cmet.2008.07.005

[25] Ursini F, Maiorino M. Lipid peroxidation and ferroptosis: the role of GSH and GPx4. Free Radic Biol Med. 2020;152:175–85. 32165281 10.1016/j.freeradbiomed.2020.02.027

[26] Rochette L, Dogon G, Rigal E, Zeller M, Cottin Y, Vergely C. Lipid peroxidation and iron metabolism: two corner stones in the homeostasis control of ferroptosis. Int J Mol Sci. 2022;24(1):449. 10.3390/ijms24010449. 10.3390/ijms24010449 PMC9820499 36613888

[27] Shrestha RK, Nassar ZD, Hanson AR, Iggo R, Townley SL, Dehairs J, et al. ACSM1 and ACSM3 regulate fatty acid metabolism to support prostate cancer growth and constrain ferroptosis. Cancer Res. 2024;84(14):2313–32. 38657108 10.1158/0008-5472.CAN-23-1489

[28] Yuan Y, Xu J, Jiang Q, Yang C, Wang N, Liu X, et al. Ficolin 3 promotes ferroptosis in HCC by downregulating IR/SREBP axis-mediated MUFA synthesis. J Exp Clin Cancer Res. 2024;43(1):133. 38698462 10.1186/s13046-024-03047-2 PMC11067213

[29] Li P, Lin Q, Sun S, Yang N, Xia Y, Cao S, et al. Inhibition of cannabinoid receptor type 1 sensitizes triple-negative breast cancer cells to ferroptosis via regulating fatty acid metabolism. Cell Death Dis. 2022;13(9):808. 36130940 10.1038/s41419-022-05242-5 PMC9492666

[30] Eid RA, Soltan MA, Eldeen MA, Shati AA, Dawood SA, Eissa M, et al. Assessment of RACGAP1 as a prognostic and immunological biomarker in multiple human tumors: a multiomics analysis. Int J Mol Sci. 2022;23(22):14102. 10.3390/ijms232214102. 10.3390/ijms232214102 PMC9695706 36430577

[31] Lin J, Zhu Y, Lin Z, Yu J, Lin X, Lai W, et al. The expression regulation and cancer-promoting roles of RACGAP1. Biomolecules. 2024;5(1):3. 10.3390/biom15010003. 10.3390/biom15010003 PMC11760467 39858398

[32] Zhou D, Ren K, Wang M, Wang J, Li E, Hou C, et al. Long non-coding RNA RACGAP1P promotes breast cancer invasion and metastasis via miR-345-5p/RACGAP1-mediated mitochondrial fission. Mol Oncol. 2021;15(2):543–59. 33252198 10.1002/1878-0261.12866 PMC7858103

[33] Şahin S, Işık Gönül İ, Çakır A, Seçkin S, Uluoğlu Ö. Clinicopathological significance of the proliferation markers Ki67, RacGAP1, and topoisomerase 2 alpha in breast cancer. Int J Surg Pathol. 2016;24(7):607–13. 27284123 10.1177/1066896916653211

[34] Sun S, Gao T, Pang B, Su X, Guo C, Zhang R. RNA binding protein NKAP protects glioblastoma cells from ferroptosis by promoting SLC7A11 mRNA splicing in an m6A-dependent manner. Cell Death Dis. 2022;13(1):73. 35064112 10.1038/s41419-022-04524-2 PMC8783023

[35] Ngo J, Choi DW, Stanley IA, Stiles L, Molina AJA, Chen PH, et al. Mitochondrial morphology controls fatty acid utilization by changing CPT1 sensitivity to malonyl-CoA. EMBO J. 2023;42(11):e111901. 36917141 10.15252/embj.2022111901 PMC10233380

[36] Tamás SX, Roux BT, Vámosi B, Dehne FG, Török A, Fazekas L, et al. A genetically encoded sensor for visualizing leukotriene B4 gradients in vivo. Nat Commun. 2023;14(1):4610. 37528073 10.1038/s41467-023-40326-6 PMC10393954

[37] Guo W, Jing W. N-acetyl-L-cysteine reduces cervical carcinogenesis by promoting apoptosis. Drugs R D. 2023;23(2):165–74. 37266883 10.1007/s40268-023-00423-9 PMC10293158

[38] Patil R, Mohanty B, Liu B, Chandrashekaran IR, Headey SJ, Williams ML, et al. A ligand-induced structural change in fatty acid-binding protein 1 is associated with potentiation of peroxisome proliferator-activated receptor α agonists. J Biol Chem. 2019;294(10):3720–34. 30598509 10.1074/jbc.RA118.006848 PMC6416440

[39] He M, Xu S, Yan F, Ruan J, Zhang X. Fatty acid metabolism: a new perspective in breast cancer precision therapy. Frontiers in Bioscience-Landmark. 2023;28(12):348. 10.31083/j.fbl2812348 38179746

[40] Pham DV, Park PH. Adiponectin triggers breast cancer cell death via fatty acid metabolic reprogramming. J Exp Clin Cancer Res. 2022;41(1):9. 34986886 10.1186/s13046-021-02223-y PMC8729140

[41] Li R, Li X, Zhao J, Meng F, Yao C, Bao E, et al. Mitochondrial STAT3 exacerbates LPS-induced sepsis by driving CPT1a-mediated fatty acid oxidation. Theranostics. 2022;12(2):976–98. 34976224 10.7150/thno.63751 PMC8692896

[42] Liang K. Mitochondrial CPT1A: insights into structure, function, and basis for drug development. Front Pharmacol. 2023;14:1160440. 37033619 10.3389/fphar.2023.1160440 PMC10076611

[43] Dong J, Li M, Peng R, Zhang Y, Qiao Z, Sun N. Acaca reduces lipid accumulation through dual regulation of lipid metabolism and mitochondrial function via AMPK- PPARα- CPT1A axis. J Transl Med. 2024;22(1):196. 38395901 10.1186/s12967-024-04942-0 PMC10885411

[44] Lee JY, Nam M, Son HY, Hyun K, Jang SY, Kim JW, et al. Polyunsaturated fatty acid biosynthesis pathway determines ferroptosis sensitivity in gastric cancer. Proc Natl Acad Sci USA. 2020;117(51):32433–42. 33288688 10.1073/pnas.2006828117 PMC7768719

[45] Xiao Q, Lan Z, Zhang S, Ren H, Wang S, Wang P, et al. Overexpression of ZNF488 supports pancreatic cancer cell proliferation and tumorigenesis through inhibition of ferroptosis via regulating SCD1-mediated unsaturated fatty acid metabolism. Biol Direct. 2023;18(1):77. 37986084 10.1186/s13062-023-00421-6 PMC10658979

[46] Li D, Li Y. The interaction between ferroptosis and lipid metabolism in cancer. Signal Transduct Target Ther. 2020;5(1):108. 32606298 10.1038/s41392-020-00216-5 PMC7327075

[47] Yang XM, Cao XY, He P, Li J, Feng MX, Zhang YL, et al. Overexpression of Rac GTPase activating protein 1 contributes to proliferation of cancer cells by reducing Hippo signaling to promote cytokinesis. Gastroenterology. 2018;155(4):1233–e12491222. 30009820 10.1053/j.gastro.2018.07.010

[48] Ren K, Zhou D, Wang M, Li E, Hou C, Su Y, et al. RACGAP1 modulates ECT2-dependent mitochondrial quality control to drive breast cancer metastasis. Exp Cell Res. 2021;400(1):112493. 33485843 10.1016/j.yexcr.2021.112493

[49] Mao C, Wang M, Zhuang L, Gan B. Metabolic cell death in cancer: ferroptosis, cuproptosis, disulfidptosis, and beyond. Protein Cell. 2024;15(9):642–60. 38428031 10.1093/procel/pwae003 PMC11365558

[50] Hänggi K, Ruffell B. Cell death, therapeutics, and the immune response in cancer. Trends Cancer. 2023;9(5):381–96. 36841748 10.1016/j.trecan.2023.02.001 PMC10121860

[51] Hua Y, Yang S, Zhang Y, Li J, Wang M, Yeerkenbieke P, et al. Modulating ferroptosis sensitivity: environmental and cellular targets within the tumor microenvironment. J Exp Clin Cancer Res. 2024;43(1):19. 38217037 10.1186/s13046-023-02925-5 PMC10787430

[52] Xue X, Wang M, Cui J, Yang M, Ma L, Kang R, et al. Glutathione metabolism in ferroptosis and cancer therapy. Cancer Lett. 2025;621:217697. 40189013 10.1016/j.canlet.2025.217697

[53] Huang G, Cai Y, Ren M, Zhang X, Fu Y, Cheng R et al. Salidroside sensitizes Triple-negative breast cancer to ferroptosis by SCD1-mediated lipogenesis and NCOA4-mediated ferritinophagy. J Adv Res. 2024;74:589–607. 10.1016/j.jare.2024.09.027 PMC12302663 39353532

[54] Zhang S, Guo L, Tao R, Liu S. Ferroptosis-targeting drugs in breast cancer. J Drug Target. 2025;33(1):42–59. 39225187 10.1080/1061186X.2024.2399181

[55] Zhao Y, Liu Z, Liu G, Zhang Y, Liu S, Gan D, et al. Neutrophils resist ferroptosis and promote breast cancer metastasis through aconitate decarboxylase 1. Cell Metabol. 2023;35(10):1688–e17031610. 10.1016/j.cmet.2023.09.004 PMC10558089 37793345

[56] Liu Y, Chen S, Wan X, Wang R, Luo H, Chang C, et al. Tryptophan 2,3-dioxygenase-positive matrix fibroblasts fuel breast cancer lung metastasis via kynurenine-mediated ferroptosis resistance of metastatic cells and T cell dysfunction. Cancer Commun. 2024;44(11):1261–86. 10.1002/cac2.12608 PMC11570772 39221971

[57] Ma Y, Temkin SM, Hawkridge AM, Guo C, Wang W, Wang XY, et al. Fatty acid oxidation: an emerging facet of metabolic transformation in cancer. Cancer Lett. 2018;435:92–100. 30102953 10.1016/j.canlet.2018.08.006 PMC6240910

[58] Houten SM, Violante S, Ventura FV, Wanders RJ. The biochemistry and physiology of mitochondrial fatty acid β-oxidation and its genetic disorders. Annu Rev Physiol. 2016;78:23–44. 26474213 10.1146/annurev-physiol-021115-105045

[59] Li J, Xia Q, Di C, Li C, Si H, Zhou B, et al. Tumor Cell-Intrinsic CD96 mediates chemoresistance and cancer stemness by regulating mitochondrial fatty acid β-Oxidation. Adv Sci (Weinheim Baden-Wurttemberg Germany). 2023;10(7):e2202956. 10.1002/advs.202202956 PMC9982582 36581470

[60] Liu Y, Zhang Y, Xiang Q, Wu S, Zhang M, Zhou H, et al. Comprehensive characterization of fatty acid oxidation in triple-negative breast cancer: focus on biological roles and drug modulation. Eur J Pharmacol. 2025;991:177343. 39900330 10.1016/j.ejphar.2025.177343

[61] Ruidas B, Sur TK, Das Mukhopadhyay C, Sinha K, Som Chaudhury S, Sharma P, et al. Quercetin: a silent retarder of fatty acid oxidation in breast cancer metastasis through steering of mitochondrial CPT1. Breast cancer (Tokyo. Japan). 2022;29(4):748–60. 10.1007/s12282-022-01356-y 35511410

[62] Schlaepfer IR, Joshi M. CPT1A-mediated fat oxidation, mechanisms, and therapeutic potential. Endocrinology. 2020;161(2):bqz046. 10.1210/endocr/bqz046. 10.1210/endocr/bqz046 31900483

[63] Das M, Giannoudis A, Sharma V. The role of CPT1A as a biomarker of breast cancer progression: a bioinformatic approach. Sci Rep. 2022;12(1):16441. 36180554 10.1038/s41598-022-20585-x PMC9525709

[64] Han S, Wei R, Zhang X, Jiang N, Fan M, Huang JH, et al. CPT1A/2-mediated FAO enhancement-a metabolic target in radioresistant breast cancer. Front Oncol. 2019;9:1201. 31803610 10.3389/fonc.2019.01201 PMC6873486

[65] Chen Y, Zhu X, Wang J, Hu J, Zhang J, Zhang X, et al. MAZ promotes tumor proliferation and immune evasion in lung adenocarcinoma. Oncogene. 2024;43(50):3619–32. 39424990 10.1038/s41388-024-03194-y

[66] Zeng J, Zhang L, Huang L, Yu X, Han L, Zheng Y, et al. MAZ promotes thyroid cancer progression by driving transcriptional reprogram and enhancing ERK1/2 activation. Cancer Lett. 2024;602:217201. 39197582 10.1016/j.canlet.2024.217201

[67] He J, Wang J, Li T, Chen K, Li S, Zhang S. *SIPL1*, regulated by MAZ, promotes tumor progression and predicts poor survival in human Triple-Negative breast cancer. Front Oncol. 2021;11:766790. 34976812 10.3389/fonc.2021.766790 PMC8718759

[68] Li Y, Lin Y, Tang Y, Jiang M, Chen X, Chen H, et al. MAZ-mediated up-regulation of BCKDK reprograms glucose metabolism and promotes growth by regulating glucose-6-phosphate dehydrogenase stability in triple-negative breast cancer. Cell Death Dis. 2024;15(7):516. 39025830 10.1038/s41419-024-06835-y PMC11258276

