# Supplementary material for: Inhibition of RACGAP1 sensitizes triple-negative breast cancer cells to ferroptosis by regulating CPT1A-dependent fatty acid metabolism
Source: J Exp Clin Cancer Res. 2025 Dec 24;44:323. doi: 10.1186/s13046-025-03568-4 (PMC12729191; doi:10.1186/s13046-025-03568-4)
Supplement: Supplementary file 1 — Supplementary Material 1 [file 13046_2025_3568_MOESM1_ESM.docx]

**Table S2. The used sequences for primers in RT-qPCR.**

| **Species** | **Name** | **Sequences (5’-3’)** |
| --- | --- | --- |
| Human | RACGAP1-F | CTTTCCTTGCCTCTGGAGTATT |
|  | RACGAP1-R | ATCTGGTGTCTGTGGTGTTG |
|  | CPT1A-F | CTGCCTTTACGTGGTGTCTAA |
|  | CPT1A-R | GGACACGTACTCTGGGTTATTC |
|  | CPT1B-F | CTGAGACTGTGCGTTCCTGT |
|  | CPT1B-R | GCAGGTCTGCTTTTGTGTGG |
|  | ACADVl-F | AAGCTCGCGGCTCACG |
|  | ACADVl-R | TCAGAGGGGTGGGAATCTGA |
|  | HADH-F | GGCCTCGGCCAAGAAGATAA |
|  | HADH-R | GCCAGGATGTCCTCTGTCTG |
|  | FASN-F | CCTGGCTGCCTACTACATCG |
|  | FASN-R | CACATTTCAAAGGCCACGCA |
|  | ACSl1-F | CTTTTGCAGCACTCACCACC |
|  | ACSl1-R | TCTTCGTGCACCACCACTAC |
|  | ACACA-F | GAACCATCTCCCTTGGCCC |
|  | ACACA-R | GCCCTCCTTCTCCTCCAGTA |
|  | SCD1-F | CTGGCTTGCTGATGATGTGC |
|  | SCD1-R | CGCAAGAAAGTGGCAACGAA |
|  | DGAT1-F | CAACAAGGACGGAGACGCC |
|  | DGAT1-R | TCACCACACACCAGTTCAGG |
|  | LPL-F | GGGAGTTTGGCTCCAGAGTTT |
|  | LPL-R | TGTGTCTTCAGGGGTCCTTAG |
|  | LIPE-F | AGACTGGCAACCTGAACCAC |
|  | LIPE-R | TCATGTTGTGCAGGGGTCTC |
|  | ABHD-F | GAACGACCAGACCTTGCTGA |
|  | ABHD-R | CCAAAGGGTCCTGCAATCCT |
|  | CTCF-F | CTGCCTTTACGTGGTGTCTAA |
|  | CTCF-R | GGACACGTACTCTGGGTTATTC |
|  | MAZ-F | GGAGTTCAAGAACGGCTACA |
|  | MAZ-R | CCATGGTCGGCATCTTCATA |
|  | SP1-F | GCAGGATGGTTCTGGTCAAATA |
|  | SP1-R | GCTGGAGTAGGTTTGGCATAG |
|  | GAPDH-F | CCTGCACCACCAACTGCTTA |
|  | GAPDH-R | TGAGTCCTTCCACGATACCA |
| Mouse | RACGAP1-F | CCTGCACCACCAACTGCTTA |
|  | RACGAP1-R | TGAGTCCTTCCACGATACCA |
|  | CPT1A-F | GAAGTGTCGGCAGACCTATTT |
|  | CPT1A-R | GTCCTCCTCTCTATATCCCTGTT |
|  | CPT1B-F | TCCAAACGTCACTGCCTAAG |
|  | CPT1B-R | CCAATGTCTCCATGCGGTAATA |
|  | ACADVl-F | CTTTGCAGGGACTCAAGGAA |
|  | ACADVl-R | CAAGCGAGCATACTGGGTATTA |
|  | HADH-F | GGGTCCGTTTGAGCTTCTT |
|  | HADH-R | CTTCTGGGCCACCAGATTATT |
|  | FASN-F | AGACCCGAACTCCAAGTTATTC |
|  | FASN-R | GCAGCTCCTTGTATACTTCTCC |
|  | ACSl1-F | GCTTGTGGATGTGGAAGAAATG |
|  | ACSl1-R | TCTTGCTGGGTCTTTCAAGTAG |
|  | ACACA-F | ACATTCCGAGCAAGGGATAAG |
|  | ACACA-R | GGGATGGCAGTAAGGTCAAA |
|  | SCD1-F | CAACTTCACCACGTTCTTCATC |
|  | SCD1-R | CCCGTCTCCAGTTCTCTTAATC |
|  | DGAT1-F | GGCCTTACTGGTTGAGTCTATC |
|  | DGAT1-R | GTTGACATCCCGGTAGGAATAA |
|  | LPL-F | AGCAGGAAGTCTGACCAATAAG |
|  | LPL-R | ATCAGCGTCATCAGGAGAAAG |
|  | LIPE-F | CATCAACCACTGTGAGGGTAAG |
|  | LIPE-R | AAGGGAGGTGAGATGGTAACT |
|  | ABHD-F | TGACAGTGATGCGGAAGAAG |
|  | ABHD-R | AGGTTGTGTCCAAGCAAGAT |
|  | CTCF-F | ACGTGGATGTGTCTGTGTATG |
|  | CTCF-R | GGCTTCGGAGGCTTCATATT |
|  | MAZ-F | GGCCCTTCAAATGTGAGAAATG |
|  | MAZ-R | CACCTTCATGTGGTCGGAAATA |
|  | SP1-F | CCAGGTGATCATGGAACTCAA |
|  | SP1-R | GGGTTGGAGATCAGAGCTATTC |
|  | GAPDH-F | AACAGCAACTCCCACTCTTC |
|  | GAPDH-R | CCTGTTGCTGTAGCCGTATT |
