# Supplementary material for: Inhibition of RACGAP1 sensitizes triple-negative breast cancer cells to ferroptosis by regulating CPT1A-dependent fatty acid metabolism
Source: J Exp Clin Cancer Res. 2025 Dec 24;44:323. doi: 10.1186/s13046-025-03568-4 (PMC12729191; doi:10.1186/s13046-025-03568-4)
Supplement: Supplementary file 2 — Supplementary Material 2 [file 13046_2025_3568_MOESM2_ESM.docx]

**Table S1. The used sequences for shRNAs.**

| **Species** | **Name** | **Sequences (5’-3’)** |
| --- | --- | --- |
| Human | sh-RACGAP1-Sense | CTAGGACGACAAGGCAACTTT |
|  | sh-RACGAP1-Antisense | AAAGTTGCCTTGTCGTCCTAG |
|  | sh-CTCF-Sense | GCAAGGCAAGAAATGCCGTTA |
|  | sh-CTCF-Antisense | TAACGGCATTTCTTGCCTTGC |
|  | sh-MAZ-Sense | CGGCCCTTCAAATGTGAGAAA |
|  | sh-MAZ-Antisense | TTTCTCACATTTGAAGGGCCG |
|  | sh-SP1-Sense | GCTGGTGGTGATGGAATACAT |
|  | sh-SP1-Antisense | ATGTATTCCATCACCACCAGC |
| Mouse | sh-RACGAP1-Sense | CCGGCAACAATAGACTGTCAA |
|  | sh-RACGAP1-Antisense | TTGACAGTCTATTGTTGCCGG |
|  | sh-CTCF-Sense | GCAGAGAAAGTAGTTGGTAAT |
|  | sh-CTCF-Antisense | ATTACCAACTACTTTCTCTGC |
|  | sh-MAZ-Sense | GATGCTGAGCTCGGCTTATAT |
|  | sh-MAZ-Antisense | ATATAAGCCGAGCTCAGCATC |
|  | sh-SP1-Sense | GCAGCAGTAATACCACCCTAA |
|  | sh-SP1-Antisense | TTAGGGTGGTATTACTGCTGC |
