# Supplementary material for: Inhibition of RACGAP1 sensitizes triple-negative breast cancer cells to ferroptosis by regulating CPT1A-dependent fatty acid metabolism
Source: J Exp Clin Cancer Res. 2025 Dec 24;44:323. doi: 10.1186/s13046-025-03568-4 (PMC12729191; doi:10.1186/s13046-025-03568-4)

Figure 3A:

MDA-MB-231:


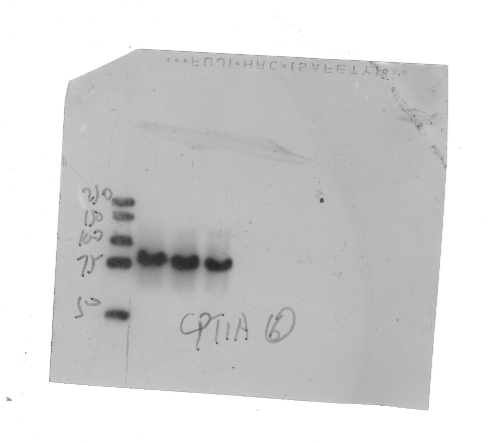


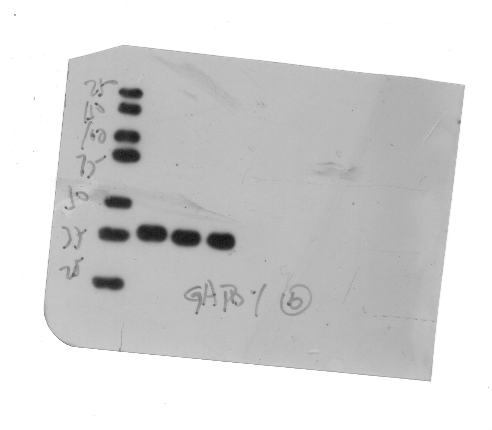


4T1:


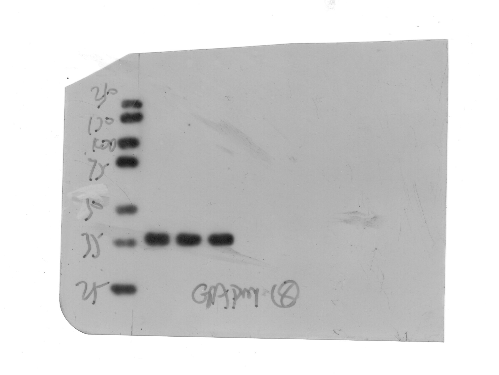

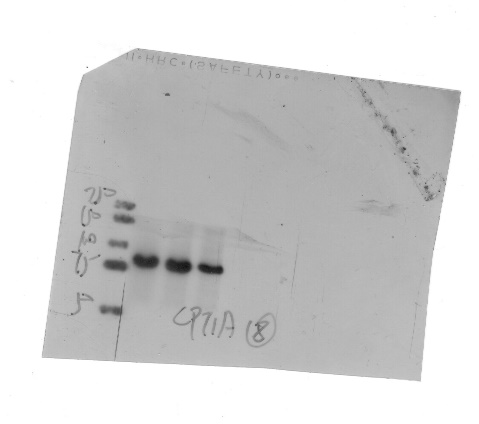


Figure 3B:


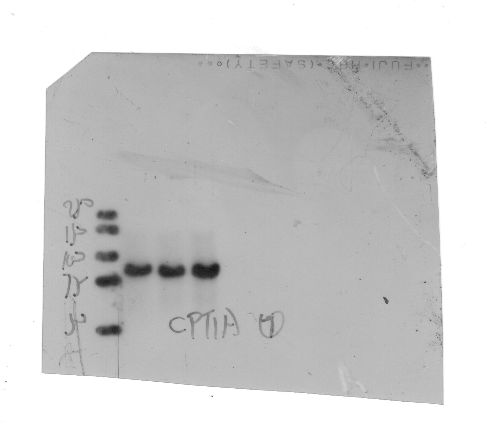
MDA-MB-231:


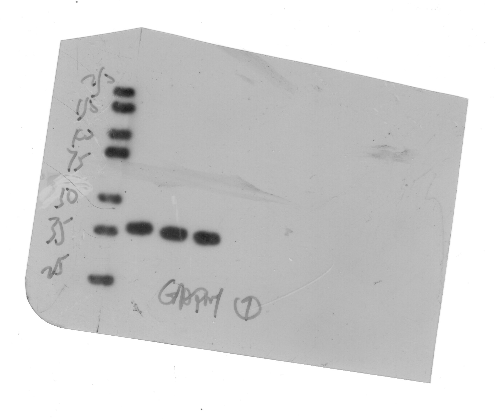


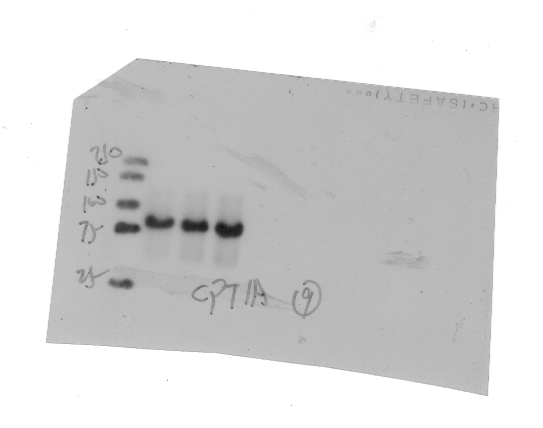
4T1:


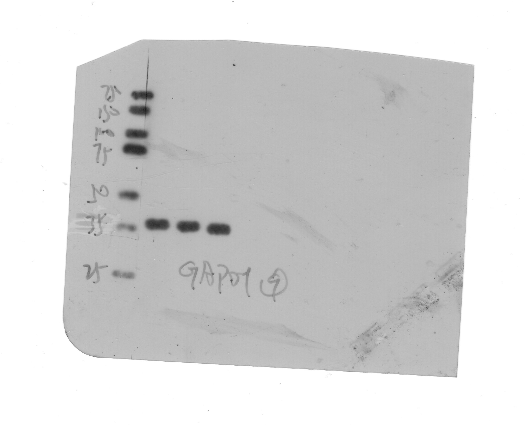


Figure 5B:

MDA-MB-231:


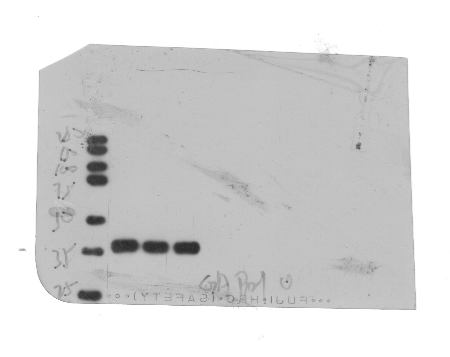

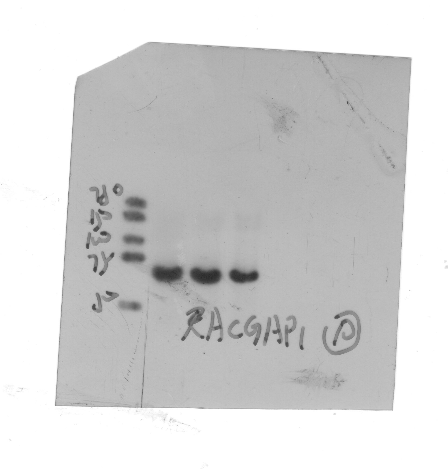


4T1:


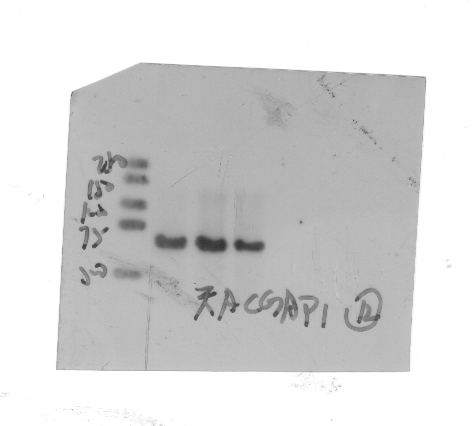


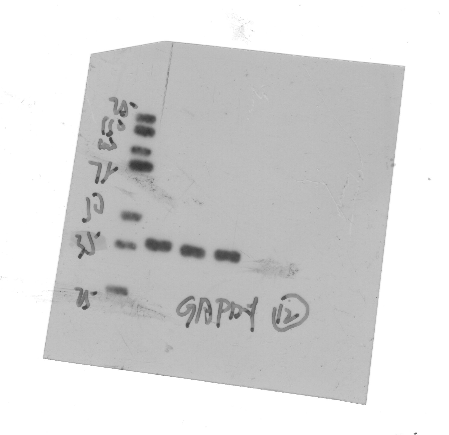


Figure 5C:

MDA-MB-231:


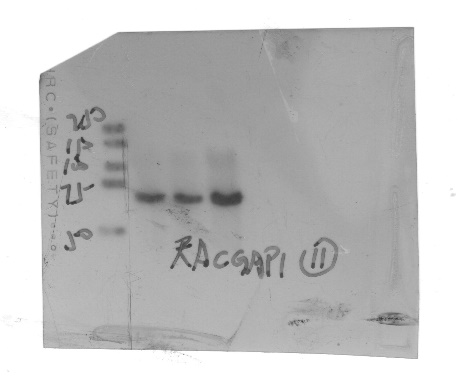


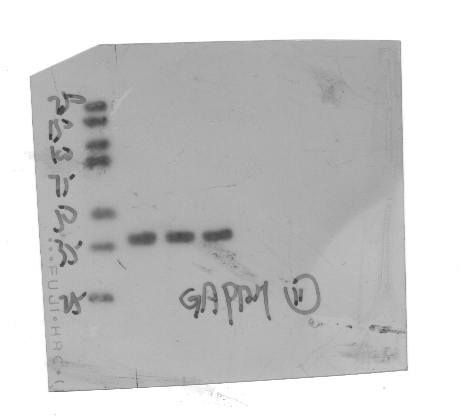


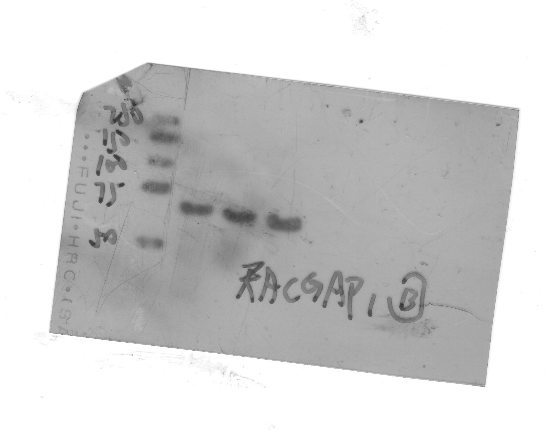
4T1:


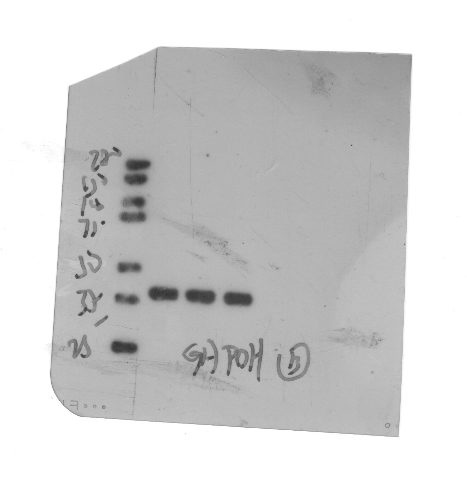


Figure 5J:

MDA-MB-231:


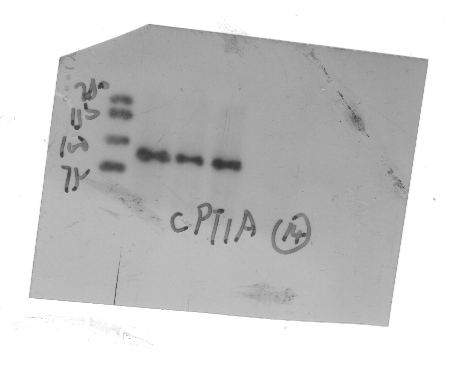


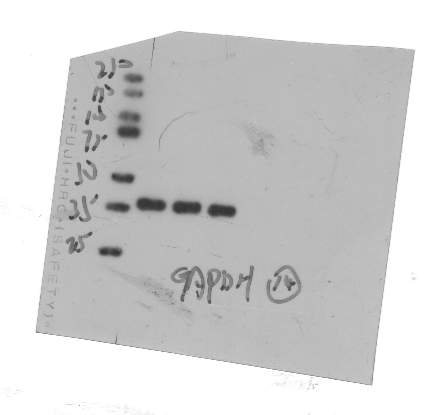


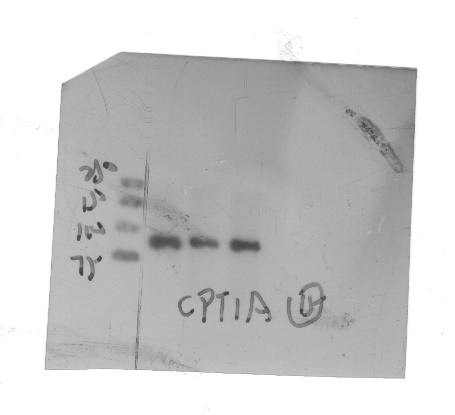
4T1:


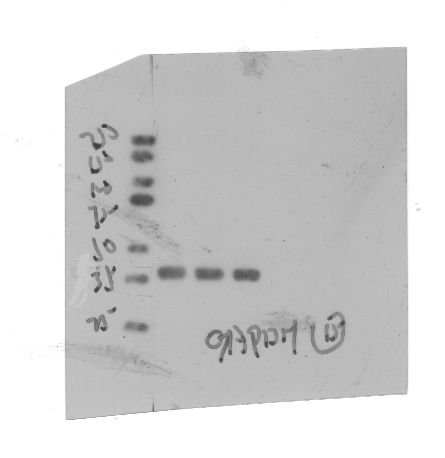


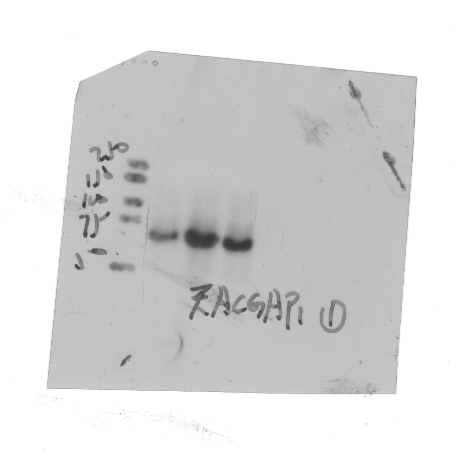
Supplementary Figure 1C:


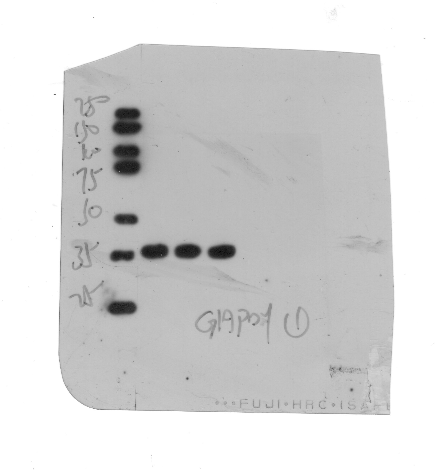


Supplementary Figure 1E:

SH- MDA-MB-231:


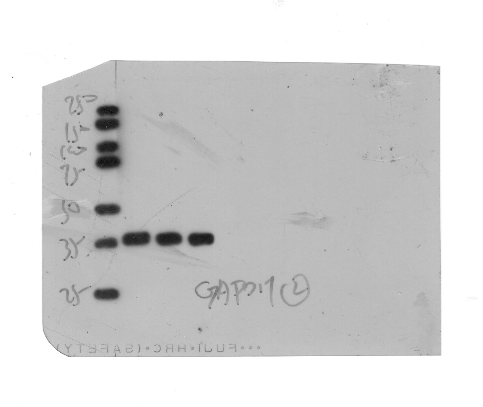

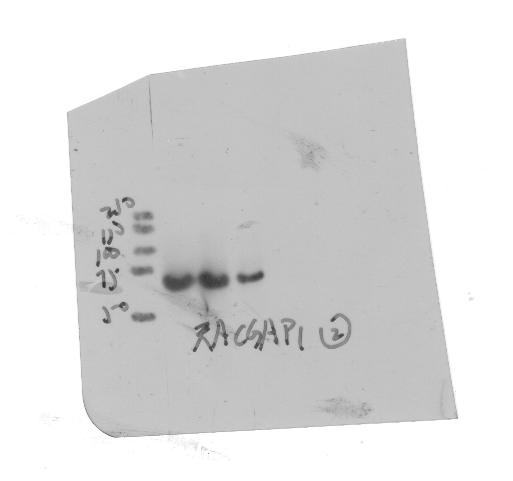


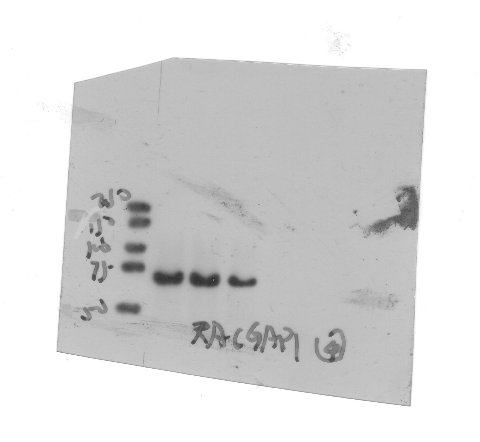
SH-4T1:


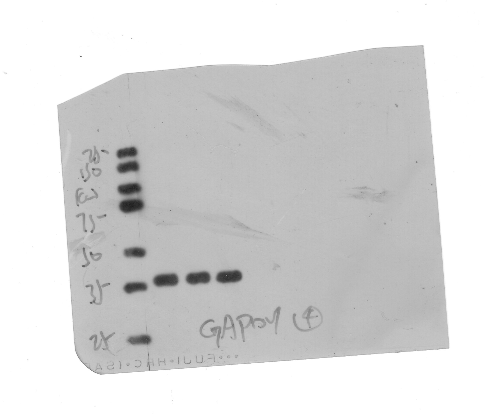


OE- MDA-MB-231:


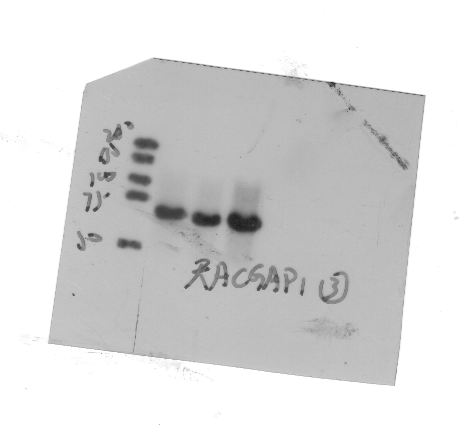


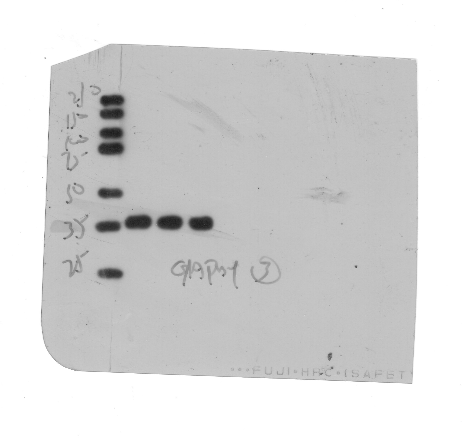


OE-4T1:


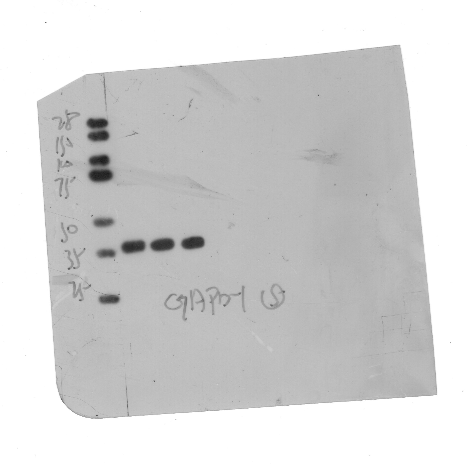

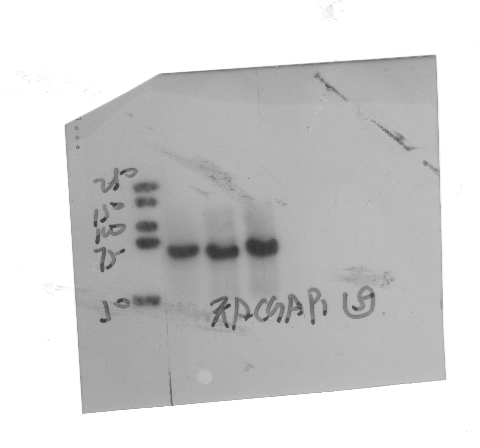


Supplementary Figure 5C:


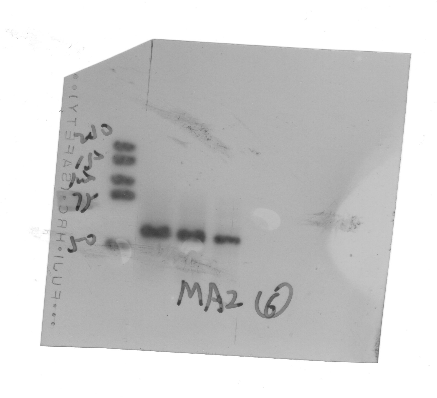
MDA-MB-231:


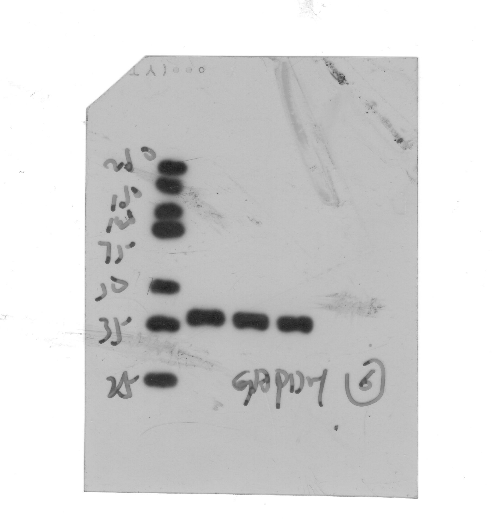


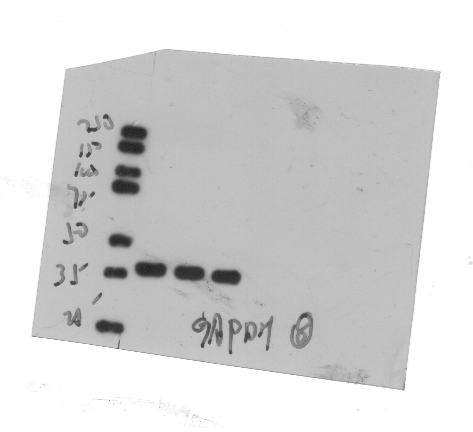

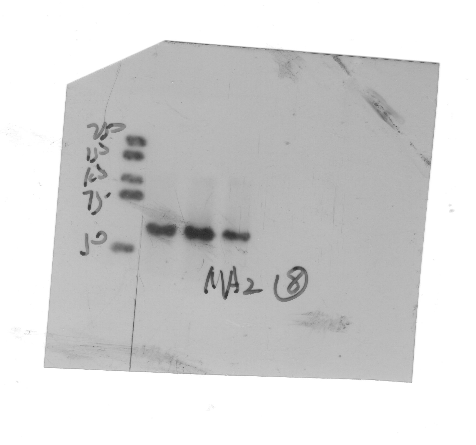
4T1:

Supplementary Figure 5D:


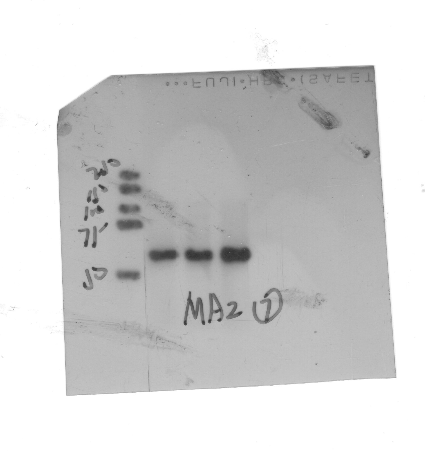
MDA-MB-231:


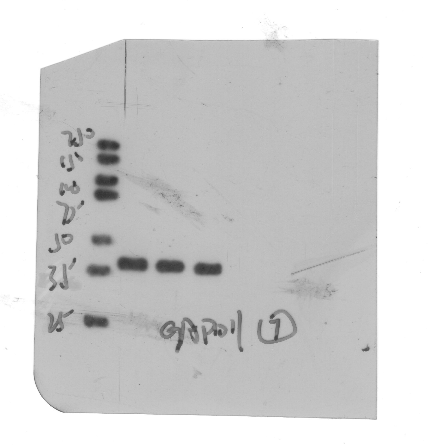


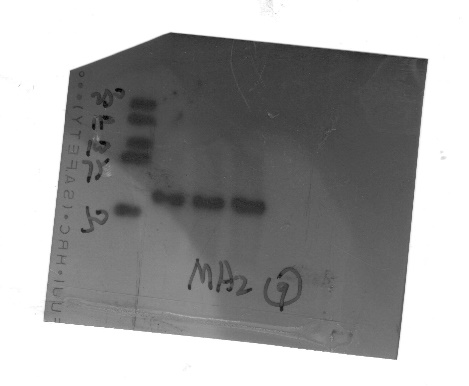
4T1:


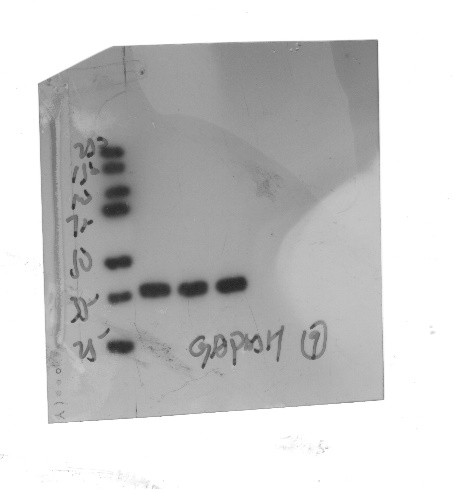

Supplement: Supplementary file 3 — Supplementary Material 3 [file 13046_2025_3568_MOESM3_ESM.docx]
