# Supplementary material for: Inhibition of RACGAP1 sensitizes triple-negative breast cancer cells to ferroptosis by regulating CPT1A-dependent fatty acid metabolism
Source: J Exp Clin Cancer Res. 2025 Dec 24;44:323. doi: 10.1186/s13046-025-03568-4 (PMC12729191; doi:10.1186/s13046-025-03568-4)
Supplement: Supplementary file 4 — Supplementary Material 4 [file 13046_2025_3568_MOESM4_ESM.docx]

**Inhibition of RACGAP1** **sensitizes triple-negative breast cancer cells to ferroptosis by regulating CPT1A-dependent fatty acid metabolism**

**Running title:** Oncogenic role of RACGAP1 in TNBC

Zhike Zhou^1#^, Ye Hua^2#^, Jun Ma^3^, Wenqiang Cong^1^, Rui Zhan^1^, Kexin Kang^1^, Lu Wang^2*^, Hongyi Wei^4*^

^1^Department of Geriatrics, The First Hospital of China Medical University, Shenyang 110001, Liaoning, PR China.

^2^Department of Urology, Shengjing Hospital of China Medical University, Shenyang, China

^3^Department of Neurobiology, School of Life Sciences, China Medical University, Shenyang, China

^4^Department of Oncology, Shengjing Hospital of China Medical University, Shenyang, China

#This author contributed equally to this work.

***Corresponding author:** Hongyi Wei

Department of Oncology, Shengjing Hospital of China Medical University, Shenyang, China, No. 36, Sanhao Street, Heping District, Shenyang 110004, Liaoning, China

Email: [hgwei@cmu.edu.cn](mailto:hgwei@cmu.edu.cn) and weihongyifeifei@163.com

**Co-corresponding author:** Lu Wang

Department of Urology, Shengjing Hospital of China Medical University, No. 36, Sanhao Street, Heping District,Shenyang 110004, Liaoning, China

Email: 872933348@qq.com

**Supplementary materials include 5 Tables and 6 Figures.**

**Figures**

**
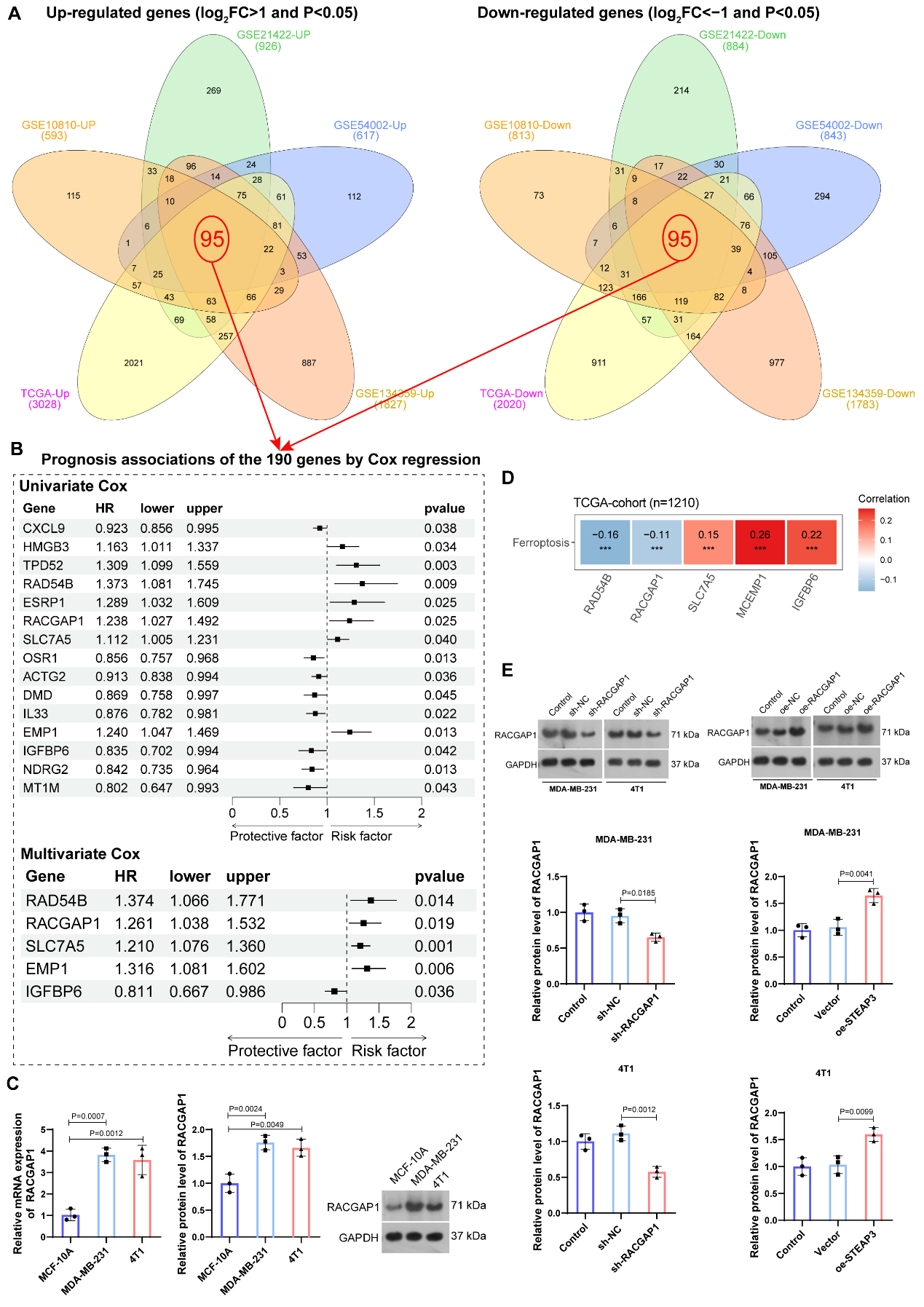
**

**Fig. S1 Expression and prognostic value of RACGAP1 in breast cancer**. A, Venn diagrams for screening of overlapped up-regulated and down-regulated genes identified from TCGA-BRCA, GSE10810, GSE54002, GSE21422 and GSE134359 datasets; B, forest plots of univariate (upper) and multivariate (below) Cox showing genes associated with survival of patients; C, mRNA and protein expression of RACGAP1 in breast cancer cell lines and mammary epithelial cells (n=3); D, correlations of prognostic genes expression with the activity of ferroptosis based on TCGA cohort (n=1210); E, validation of RACGAP1 silencing and RACGAP1 overexpression in MDA-MB-231 and 4T1 cell lines by Western blot (n=3).

**
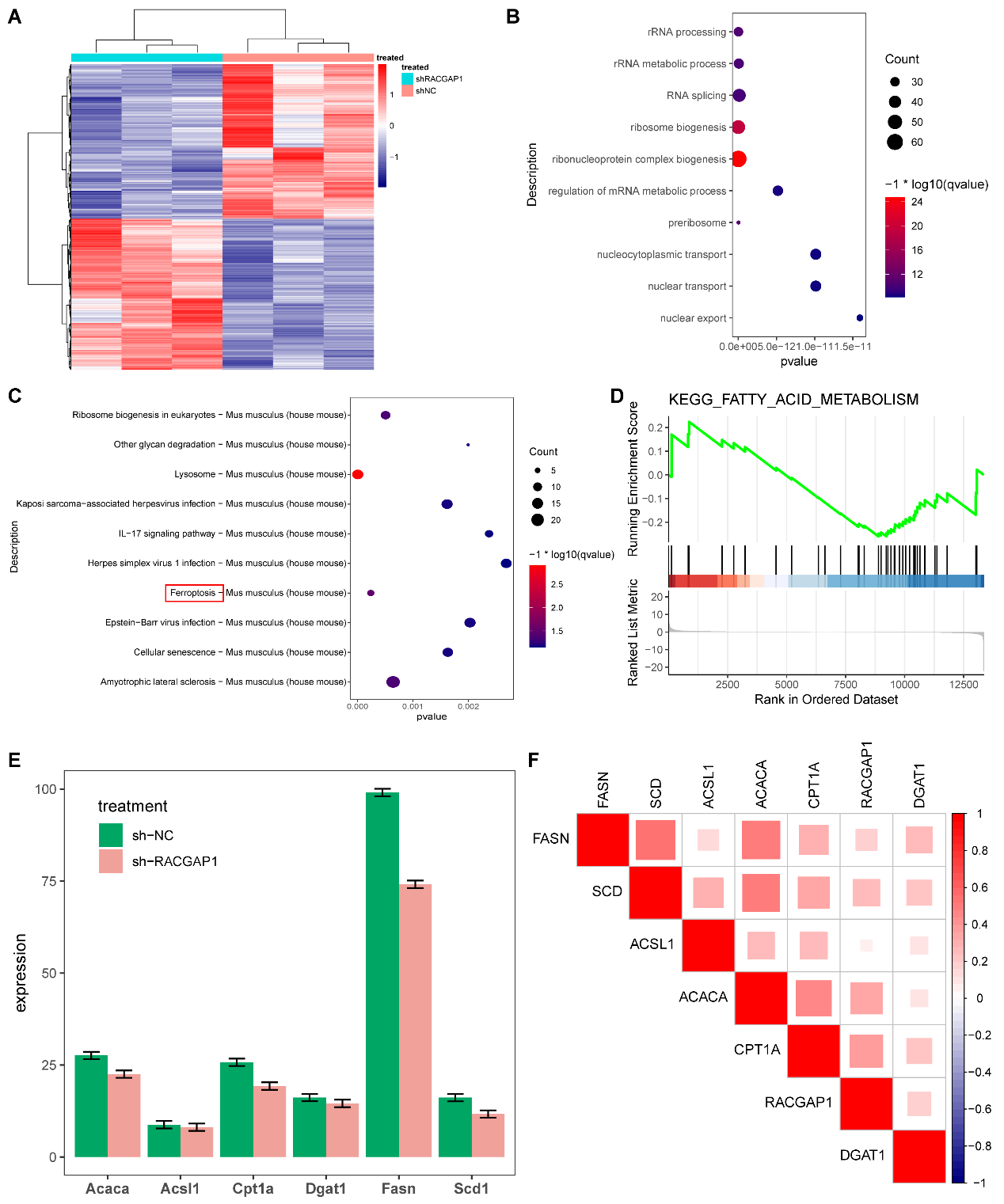
Fig. S2 RACGAP1 silencing regulated fatty acid metabolism.** A, Heatmap showing the expression pattern of differential genes between 4T1 cells transfecting with sh-RACGAP1 and sh-NC (n=3); B-C, the significantly enriched gene ontology terms and KEGG pathways for differential genes; D, gene set enrichment analysis for change on fatty acid metabolism between sh-RACGAP1 and sh-NC groups; E, expression of key genes in fatty acid metabolism pathway; F, correlation of RACGAP1 and the genes in fatty acid metabolism pathway.

**
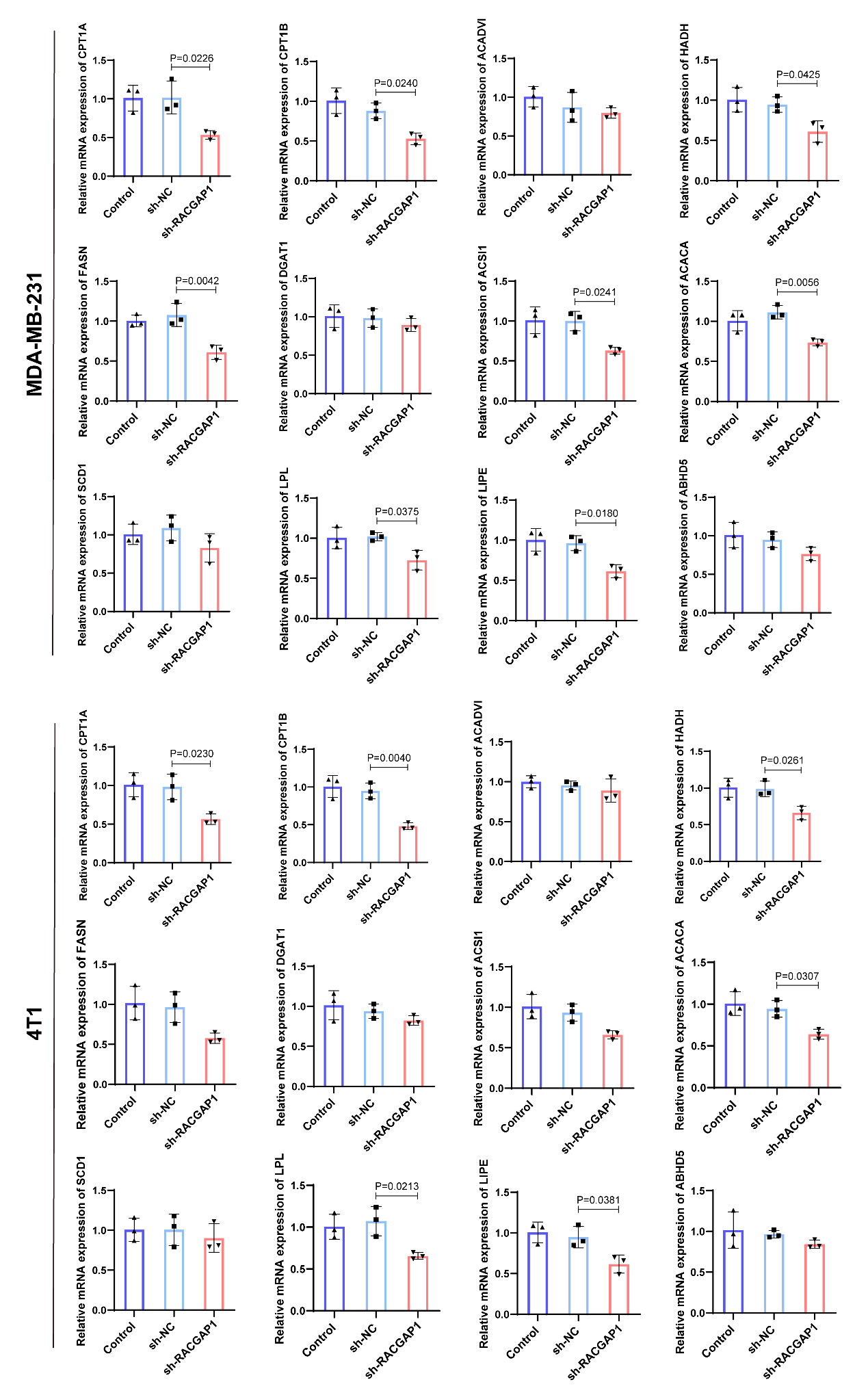
**

**Fig. S3 RACGAP1 silencing regulated the expression of genes involved in fatty acid metabolism.** The mRNA expression of genes related to FAO (CPT1A, CPT1B, ACADV1 and HADH), fatty acid synthesis (FASN, ACSl1, ACACA, SCD1 and DGAT1) and lipolysis (LP1, LIPE and ABHD5) in MDA-MB-231 and 4T1 breast cells after RACGAP1 silencing (n=3).

**
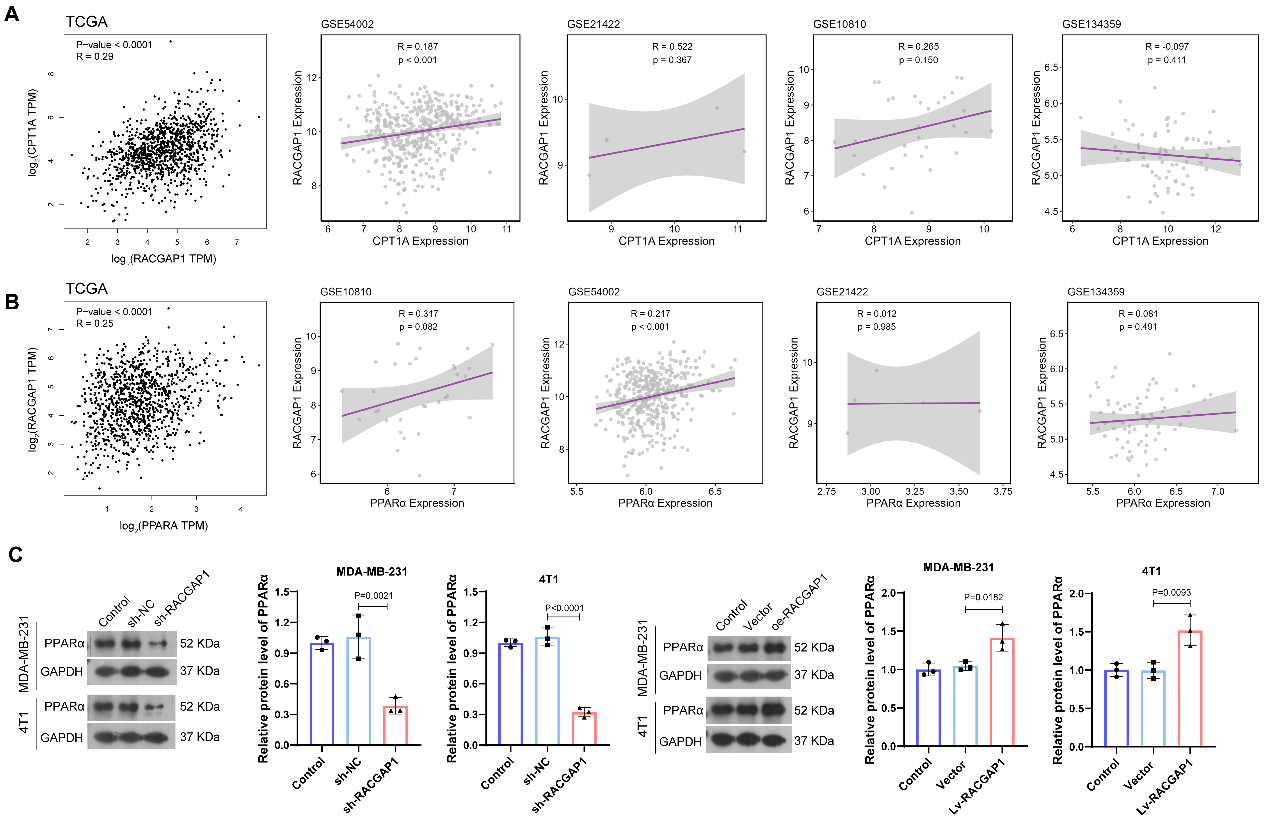
**

**Fig. S4 Correlation analysis and the regulation of RACGAP1 on PPARα expression.** A, correlations scatterplots showing the correlations between CPT1A and RACGAP1 expression in TCGA-BRCA, GSE54002, GSE21422, GSE10810 and GSE134359 datasets; B, correlations scatterplots showing the correlations between PPARα and RACGAP1 expression in TCGA-BRCA, GSE10810, GSE54002, GSE21422 and GSE134359 datasets; C, protein bands and quantification of PPARα expression in MDA-MB-231 and 4T1 cells after RACGAP1 silencing or overexpression.

**
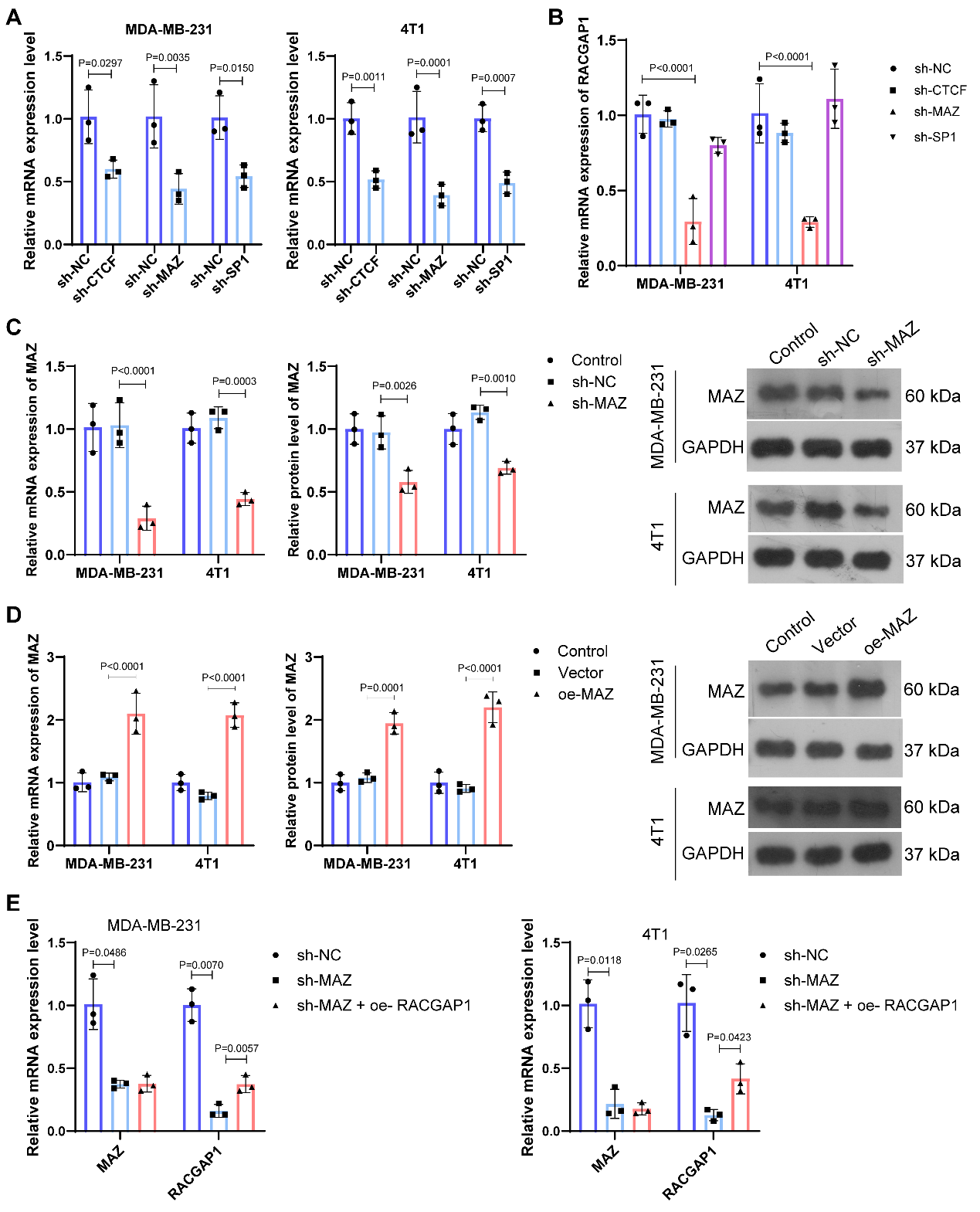
**

**Fig. S5 Validation of gene silencing and/or overexpression.** A, mRNA expression of transcription factors CTCF, MAZ and SP1 in MDA-MB-231 and 4T1 breast cells after transfecting with corresponding shRNA (n=3); B, mRNA expression of RACGAP1 in MDA-MB-231 and 4T1 breast cells after silencing of CTCF, MAZ and SP1, respectively (n=3); C-D, the mRNA and protein expression of MAZ in MDA-MB-231 and 4T1 breast cells after MAZ silencing (C) or MAZ overexpression (D) (n=3); E, mRNA expression of MAZ and RACGAP1 in MDA-MB-231 and 4T1 breast cells after MAZ silencing and/or RACGAP1 overexpression (n=3).


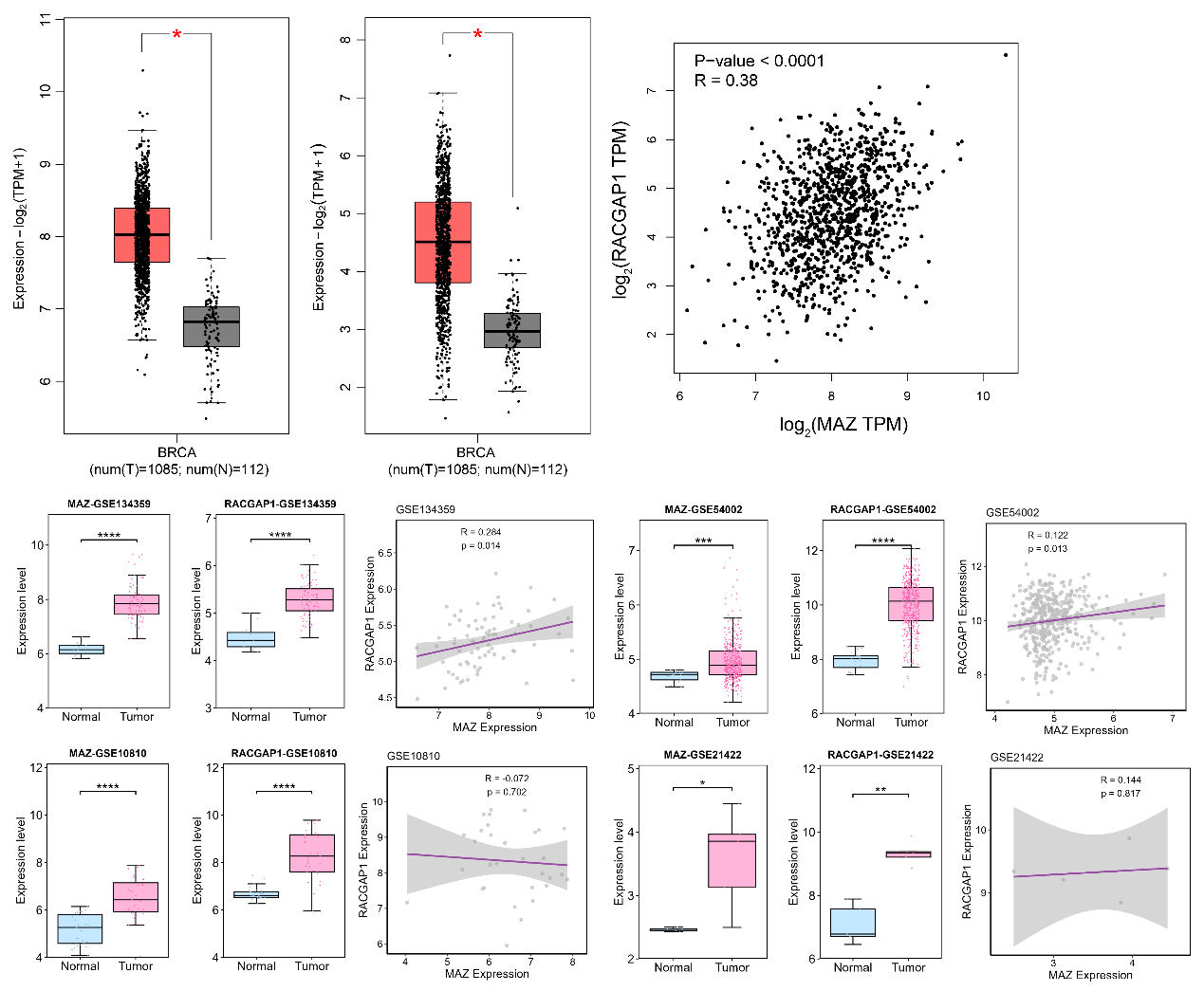


**Fig. S6 Correlation analysis between MAZ and RACGAP1.** Boxplots showing the expression of MAZ and RACGAP1 in BRCA tumor and normal tissues in TCGA-BRCA, GSE54002, GSE21422, GSE10810 and GSE134359 datasets. Correlation scatterplots showing the correlations between MAZ and RACGAP1 expression in TCGA-BRCA, GSE54002, GSE21422, GSE10810 and GSE134359 datasets.
