# Supplementary material for: Inhibition of RACGAP1 sensitizes triple-negative breast cancer cells to ferroptosis by regulating CPT1A-dependent fatty acid metabolism
Source: J Exp Clin Cancer Res. 2025 Dec 24;44:323. doi: 10.1186/s13046-025-03568-4 (PMC12729191; doi:10.1186/s13046-025-03568-4)
Supplement: Supplementary file 5 — Supplementary Material 5 [file 13046_2025_3568_MOESM5_ESM.docx]

**Table S3. The used primers in dual-luciferase assay**

|  | **Primers** | **Sequences (5'-3')** |
| --- | --- | --- |
| RACGAP1 promoter amplification | RACGAP1 | GGATCTTCCAGAGATAAGCTTCCTGGGTTCAAGCGATTCTCC |
|  |  | CTGCCGTTCGACGATCTCGAGGGGAGGAGGCTGGGGACT |
| Site-directed mutagenesis | Primer-1 (272-279) | AACTATATCCAAGCAGGGCTCCCCAGAG |
|  |  | CTGCTTGGATATAGTTATTGACATGCTG |
|  | Primer-2 (928-935) | GCCAAACAGCATAGGCTGCGAGCTGGTA |
|  |  | GCCTATGCTGTTTGGCATAAGCAACTTC |
|  | Primer-3 (929-936) | CCAAACAGCATAGGCTGCGAGCTGGTAC |
|  |  | AGCCTATGCTGTTTGGCATAAGCAACTT |
|  | Primer-4 (1786-1793) | TTGAACCTGAGGTTCCAGAAAGCCGAGA |
|  |  | GGAACCTCAGGTTCAAGCGATTTTCCCA |
|  | Primer-5 (1994-2001) | TCCCCAGCATGGATACTATGATGCTGAA |
|  |  | GTATCCATGCTGGGGACTGAAGTAGGCA |
