# Supplementary material for: Inhibition of RACGAP1 sensitizes triple-negative breast cancer cells to ferroptosis by regulating CPT1A-dependent fatty acid metabolism
Source: J Exp Clin Cancer Res. 2025 Dec 24;44:323. doi: 10.1186/s13046-025-03568-4 (PMC12729191; doi:10.1186/s13046-025-03568-4)
Supplement: Supplementary file 6 — Supplementary Material 6 [file 13046_2025_3568_MOESM6_ESM.docx]

**Table S4. The used sequences for primers in CHIP-PCR.**

| **Species** | **Name** | **Sequences (5’-3’)** |
| --- | --- | --- |
| Human | Primer1-F | CAGAAGTTAAAATGCTAG |
|  | Primer1-R | TTCCCTCCCTCTCAATC |
|  | Primer2-F | GAGCAAGACTCTGTCTCAAA |
|  | Primer2-R | TTGCCATGTTGTCCAGGCTG |
|  | Primer3-F | GGAGTTCGAGACCAGCCTG |
|  | Primer3-R | GTTTGAGCCGTCATGCCTGG |
|  | Primer4-F | AGTTTGATGAATGAATAAGG |
|  | Primer4-R | GGAAGCATAG GCTTCTCAG |
|  | Primer5-F | CAATTTCAGAGCAGGCTTCA |
|  | Primer5-R | TTTCCTCCTGCTCTGTCCTG |
| Mouse | Primer1-F | CTATGCAA GGGTGCTGAT |
|  | Primer1-R | TCCTGTGCCCAGGAGATG |
|  | Primer2-F | TAGAACACCTGCTGCACTTG |
|  | Primer2-R | CACAGAGGCCAGAAGAG |
|  | Primer3-F | AGTACTACAGACAGCTTGGT |
|  | Primer3-R | CTCTTTCACATAAATAG |
|  | Primer4-F | ATTAGTACTTGATCAGCACCCA |
|  | Primer4-R | TGTACATCTATTAAGCACCTACGACA |
|  | Primer5-F | TCTCAGACCACACCCTAGTA |
|  | Primer5-R | AATACTTGGAAACTGATTGGGCTA |
