# Supplementary figures and images for: Inhibition of RACGAP1 sensitizes triple-negative breast cancer cells to ferroptosis by regulating CPT1A-dependent fatty acid metabolism
Source: J Exp Clin Cancer Res. 2025 Dec 24;44:323. doi: 10.1186/s13046-025-03568-4 (PMC12729191; doi:10.1186/s13046-025-03568-4)

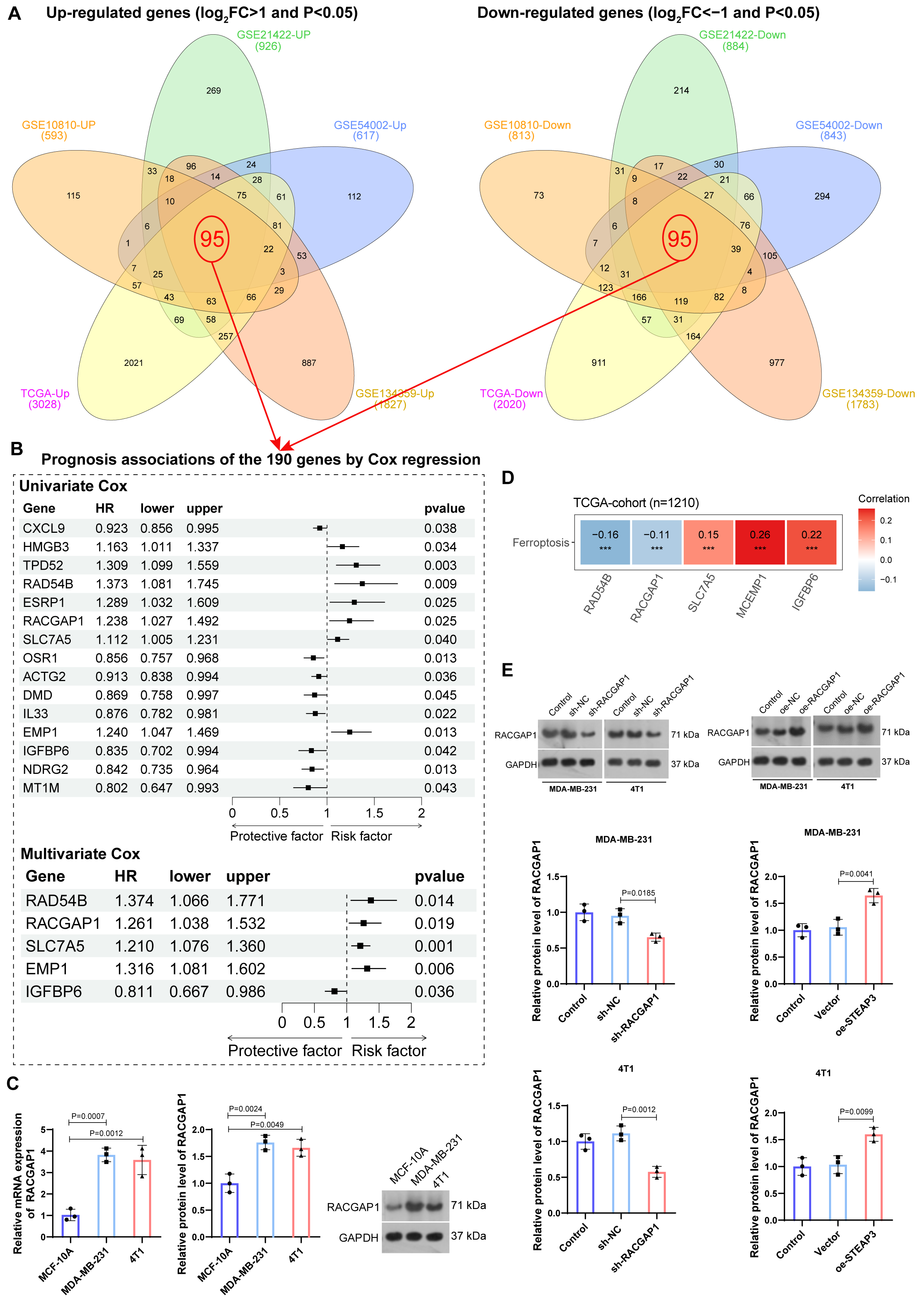

Supplement: Supplementary file 9 — Supplementary Material 9 [file 13046_2025_3568_MOESM9_ESM.tif]

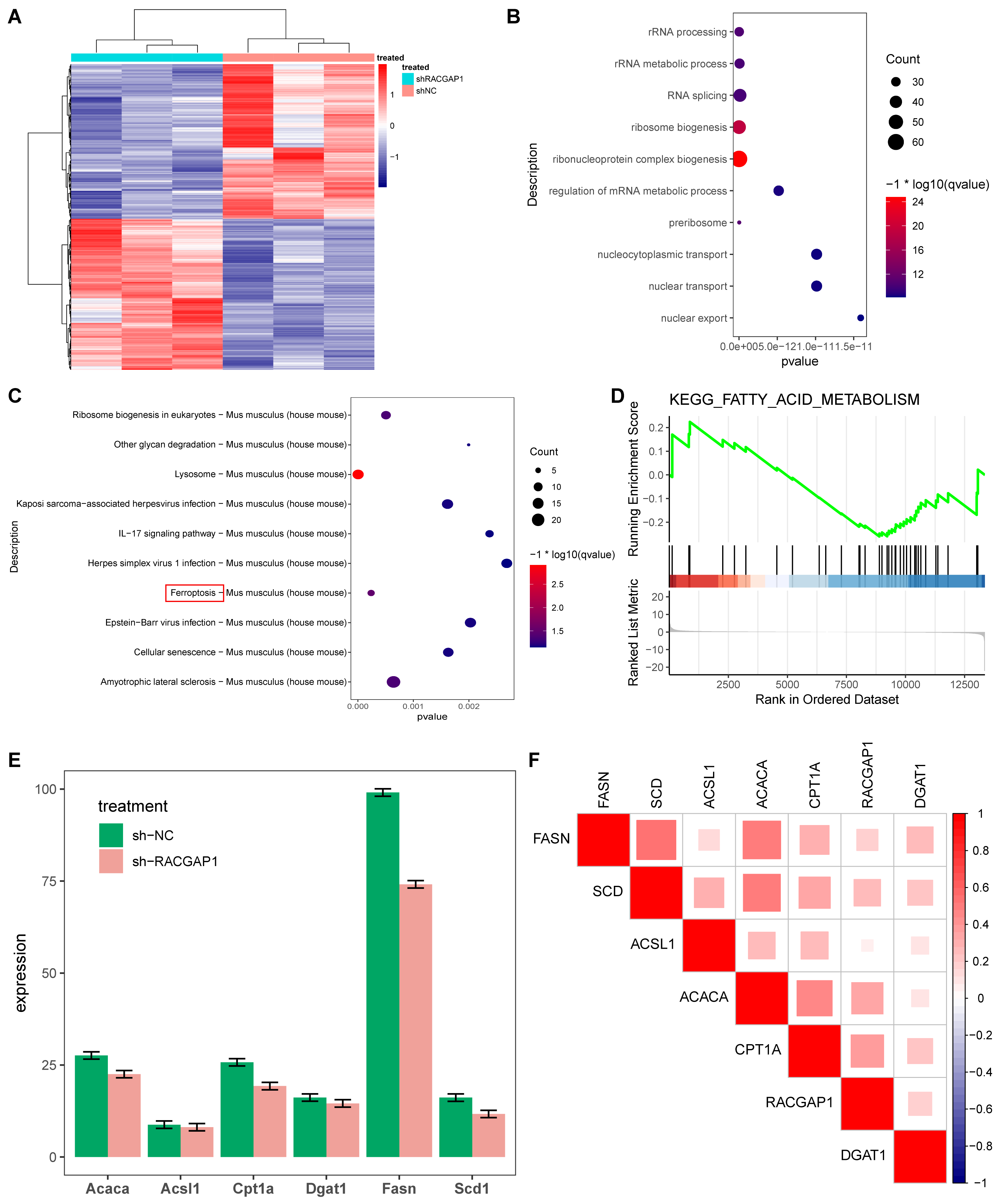

Supplement: Supplementary file 10 — Supplementary Material 10 [file 13046_2025_3568_MOESM10_ESM.tif]

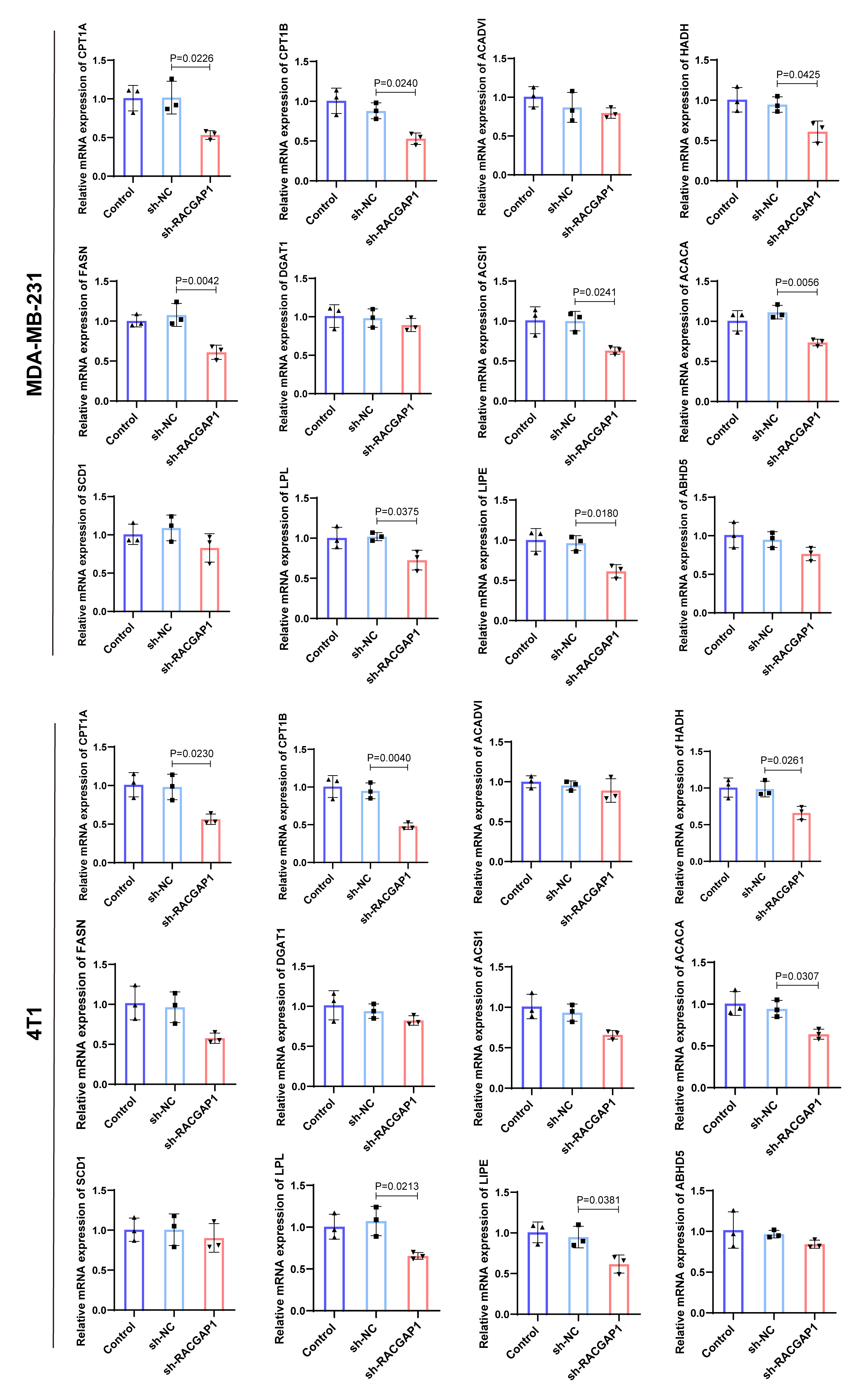

Supplement: Supplementary file 11 — Supplementary Material 11 [file 13046_2025_3568_MOESM11_ESM.tif]

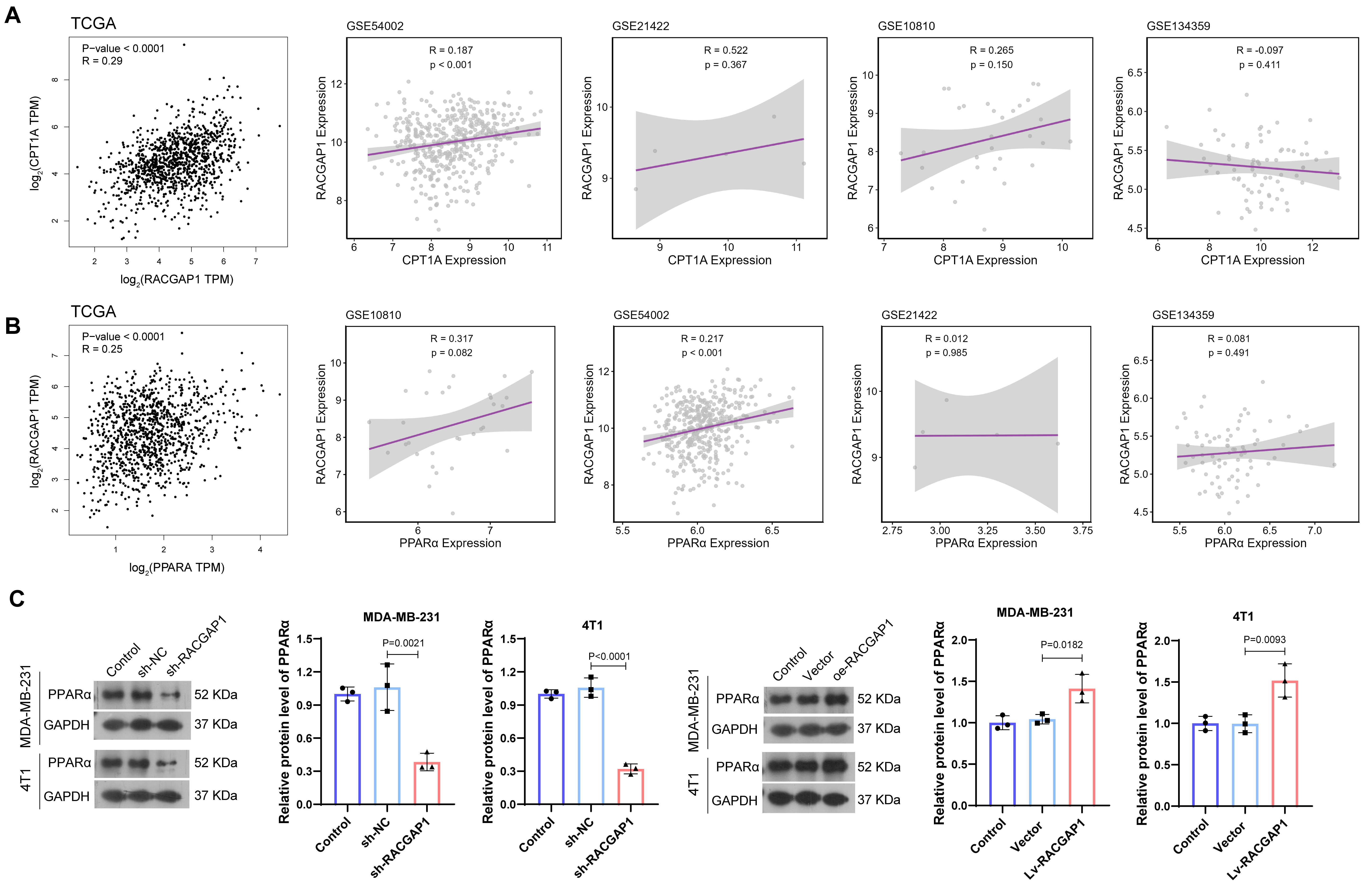

Supplement: Supplementary file 12 — Supplementary Material 12 [file 13046_2025_3568_MOESM12_ESM.tif]

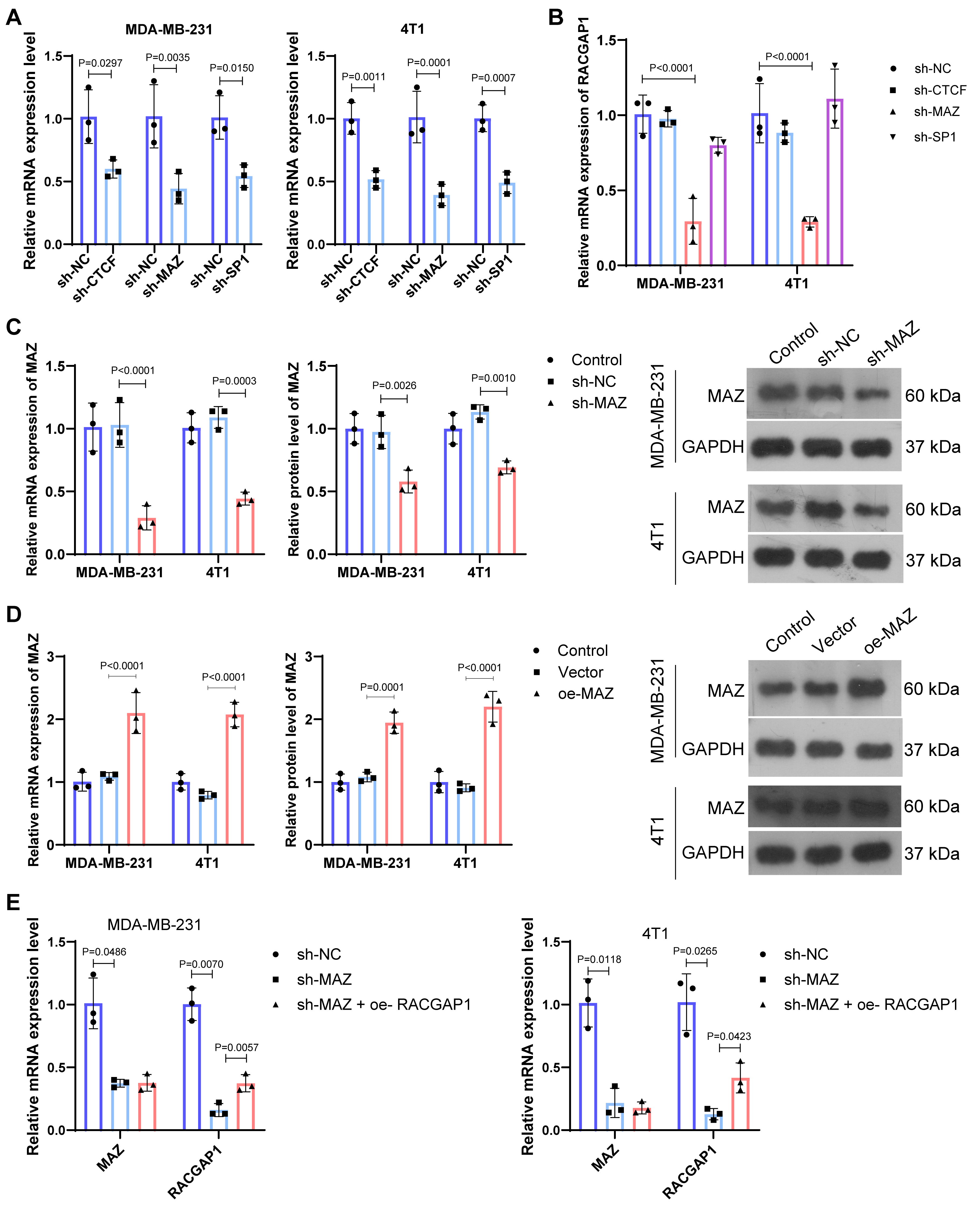

Supplement: Supplementary file 13 — Supplementary Material 13 [file 13046_2025_3568_MOESM13_ESM.tif]

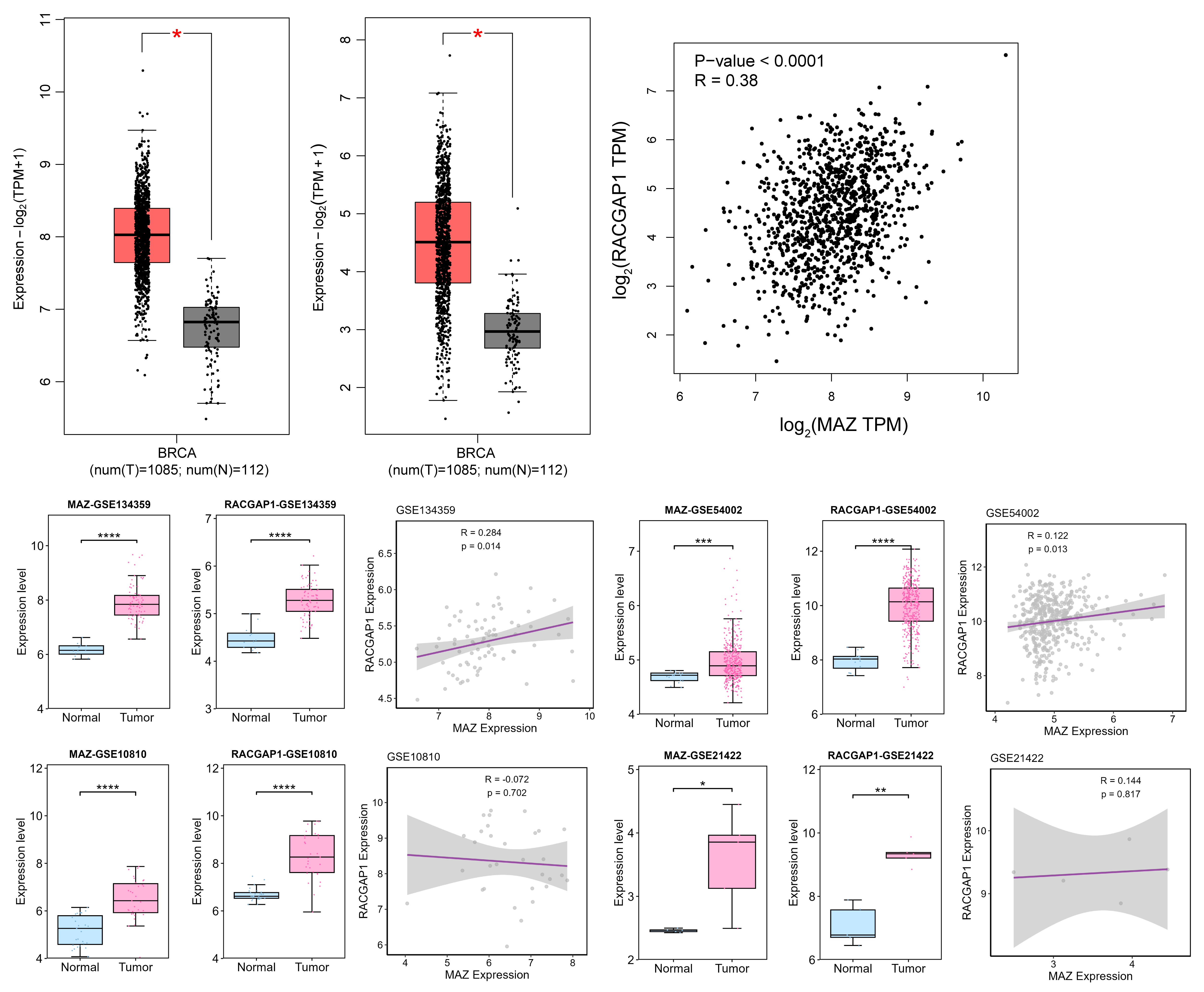

Supplement: Supplementary file 14 — Supplementary Material 14 [file 13046_2025_3568_MOESM14_ESM.tif]
